# The serine protease inhibitor HAMpin-1 produced by the ectoparasite Hyalomma anatolicum salivary gland modulates the host complement system

**DOI:** 10.1016/j.jbc.2024.107684

**Published:** 2024-08-17

**Authors:** Rajitha Mood, Krishnagaanth Mohankumar, Macha Vijay, Anand Srivastava

**Affiliations:** 1 Molecular Pathogenesis and Vector Laboratory, National Institute of Animal Biotechnology (NIAB), Hyderabad, Telangana, India; 2 Regional Centre for Biotechnology (RCB), Faridabad, India

**Keywords:** *Hyalomma anatolicum*, salivary gland extract, complement pathway, complement modulation, hemolysis, pull-down, mass spectrometry, serpin, protease inhibition

## Abstract

Ticks are notable vectors of diseases affecting both humans and animals, with *Hyalomma anatolicum* being of particular significance due to its wide distribution and capability to transmit a variety of pathogens, including *Theileriaannulata* and Crimean–Congo haemorrhagic fever virus. This study aimed to investigate the inhibitory effects of *H. anatolicum* salivary gland extract (HaSGE) and the identification of its key component on the complement system of the host's innate immune defense. We demonstrated that HaSGE exerts a dose-dependent inhibition on the complement activation in a host-specific manner. Mechanistic studies revealed that HaSGE interferes with deposition and cleavage of complement proteins C3 and C5, thus preventing the formation of the membrane attack complex. Further, we identified a serine protease inhibitor, *Hyalomma anatolicum* serpin-1 (HAMpin-1), from the HaSGE through proteomic analysis and characterized its structure, function, and interaction with complement proteins. HAMpin-1 exhibited potent inhibitory activity against chymotrypsin and cathepsin-G, and notably, it is the first serpin from ticks shown to inhibit the classical and lectin pathways of the complement system. The expression of HAMpin-1 was highest in the salivary glands, suggesting its crucial role in blood feeding and immune evasion. Our findings revealed one of the potential mechanisms used by *H. anatolicum* to modulate host immune responses at the interface, offering new insights into tick–host interactions.

Ticks, tiny blood-sucking arthropods, are the primary vector of animal pathogens and rank second only to mosquitoes in their significance for human pathogens (1). *Hyalomma anatolicum* is a widely distributed tick species found in Africa, Europe, the Middle East, and certain regions of Central Asia, such as China and India (2). This tick has a diverse host range and ability to transmit pathogens like the Crimean–Congo haemorrhagic fever virus to humans, *Theileria* spp. to cattle and horses, and *Babesia* spp. to sheep (2). Hard ticks, including *Hyalomma* species, are known for their extended attachment to hosts during feeding. Throughout this process, ticks release a wide array of molecules into the interface between tick and host *via* saliva from salivary glands. This secretion aids in countering the host response and facilitates the transmission of pathogens (3). Prior research has indicated that tick salivary protein modulates the host's complement system (4, 5, 6). However, no studies have been conducted to explore the *H*. *anatolicum* salivary gland lysate and its proteins in the species-specific complement system.

The complement system is integral to the host's innate immune system. It becomes activated during various infections and, in the case of ticks, through tick bites. The complement proteins present in plasma predominantly exhibit protease activity and, once triggered, initiate a sequence of reactions known as the triggered-enzyme cascade. Activation of the complement system can occur through three distinctive pathways: classical, alternative, and lectin. Each pathway responds to specific triggers like antigen-antibody complex, mannose-binding lectin, and nonself-surface. Regardless of initiation, all the pathways converge on activating the C3 complement protein, ultimately forming the membrane attack complex (MAC) responsible for lysing cell membranes (7). Furthermore, the cleavage of complement proteins yields small fragments that induce an inflammatory response. These responses are crucial in bridging the innate and adaptive response (8). It is worth noting that this cascade is subject to strict regulation at multiple levels to prevent self-destruction. These regulatory mechanisms include the inhibition of proteases, control of the MAC, and decay and destruction of convertase enzyme (7).

Reports suggest that ticks and other blood-feeding organisms exploit host's regulatory mechanisms to evade the complement cascade. Haematophagous arthropods, in the course of their blood-feeding process, introduce a substantial quantity of pharmacologically active compounds to modulate the host response. Some of these molecules possess anticlotting, antiplatelet aggregation, anticomplement, and vasodilatory activity (9). Although prior research has demonstrated the anticomplement effects of tick salivary gland constituents across various species such as *Rhipicephalus microplus* (5), *Ixodes scapularis* (10), *Ixodes ricinus* (11), *Amblyomma americanum* (12), and *Amblyomma cajennese* (13), an investigation into complement evasion by *H*. *anatolicum* has remained conspicuously absent. This investigation is crucial for a comprehensive understanding of the tick–host relationship in a species-specific manner and to identify potential novel candidates for vaccine development. This study delves into the species-specific anticomplement capability of *H*. *anatolicum* salivary gland lysate.

Additionally, we used a proteomic-based approach to identify salivary gland molecules that interact with distinct complement proteins. Notably, one such molecule, serpin (serine protease inhibitor) was identified, which unveiled compelling evidence of its ability to inhibit the classical and lectin complement pathway. Seventy-five percent of the proteases superfamily comprises serine protease, which governs processes like coagulation, complement activity, inflammation, and tissue remodeling. Remarkably, serpins are ubiquitously found in diverse life forms ranging from prokaryotes to eukaryotes as well as in viruses and archaea. This protein superfamily possesses a highly conserved secondary structure, including three primary beta-sheets, eight to nine helices, and a distinct reactive center loop (RCL) that protrudes from the main structure. The serpins are recognized as unique, often referred to as "suicide" or "irreversible" inhibitors, because they transform from a metastable state to a relaxed state upon binding to substrate proteases. Serpins are categorized into two types: inhibitory serpins and noninhibitory serpins. In the case of inhibitory serpins, specific proteases cleave the bait recognition sequence (P1-P1'site) located in the serpin RCL. Following cleavage, a C-terminal loop insert into the core structure. If insertion occurs rapidly, it forms a covalent complex (serpin-protease complex), which disrupts the active site of proteases and facilitates proteasomal degradation. Conversely, if the loop insertion is delayed, proteases cleave the serpins and escape the trap without forming a covalent complex, thereby no inhibition. Noninhibitory serpins serve various roles, including chaperones and hormone transporters (14, 15). In this study, we provide additional support for the tick evasion of the host complement system. We have shown species-specific inhibition of the complement cascade by *H. anatolicum* salivary gland extract (HaSGE). We also identified and characterized a novel serpin from *H. anatolicum* (named HAMpin-1) for its modulatory role in the classical and lectin complement pathways.

## Results

### HaSGE inhibits activation of host complement pathways

#### HaSGE inhibits classical and alternative complement pathways in a host-specific manner

**Figure 1:**
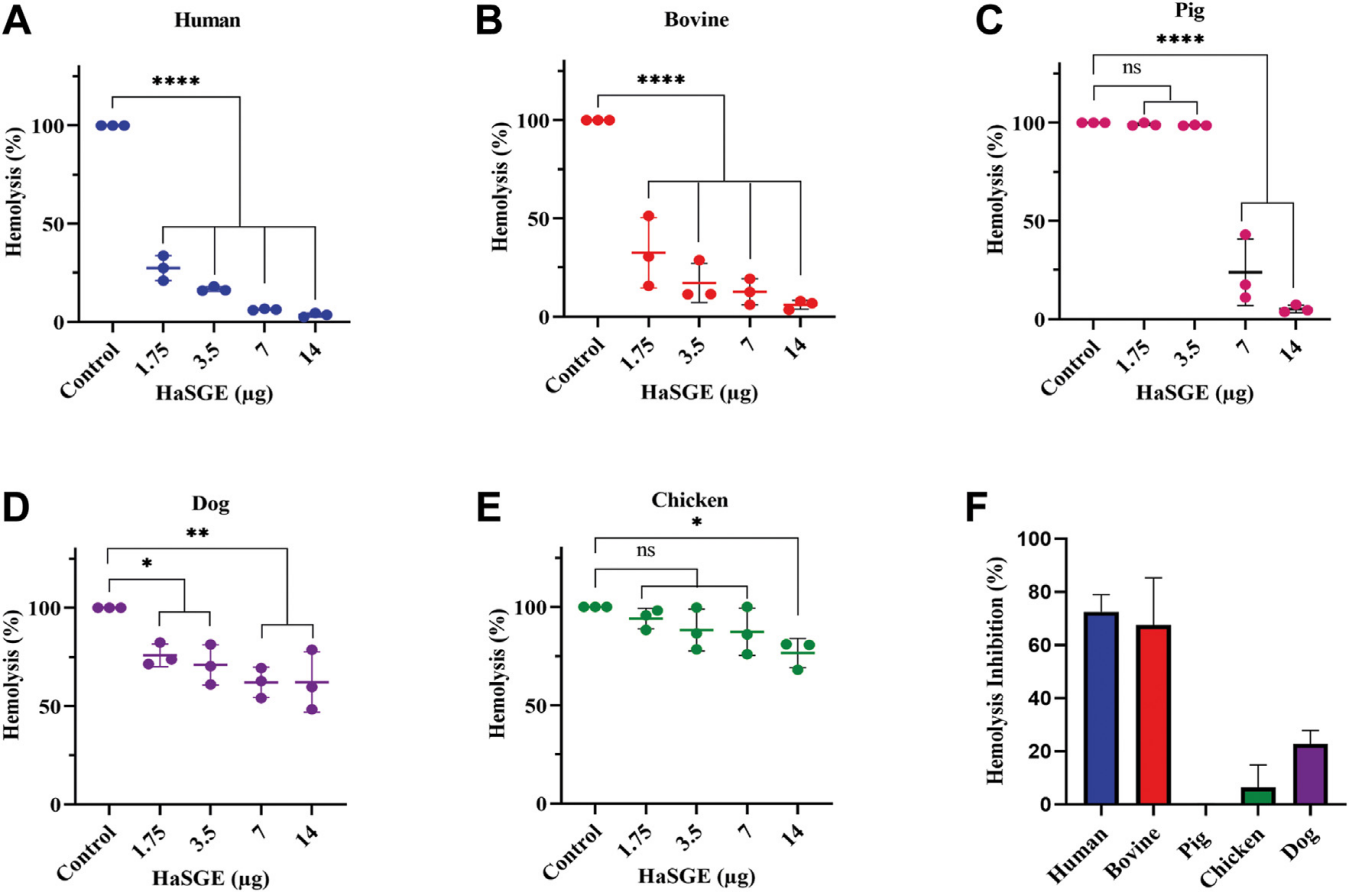
**Effect of HaSGE on the classical complement pathway using hemolysis assay with different host sera.** Human (*A*), Bovine (*B*), Pig (*C*), Dog (*D*), and Chicken (*E*). Serum was preincubated for 30 min at 37 °C with different amounts of HaSGE ranging from 1.75 to 14 μg (in 31 μl). The *X*-axis represents the different amounts of HaSGE ranging from 0 (control) to 14 μg. The *Y*-axis represents the hemolysis in percentage. *F*, hemolysis inhibition at 1.75 μg of HaSGE in different hosts. Each bar represents the hemolysis inhibition of HaSGE on different hosts. The values are expressed as the mean percentage of hemolysis/hemolysis inhibition ± SD from n = 3 independent experiments, each measured in duplicates. Statistical significance was determined using one-way ANOVA (∗*p* < 0.05; ∗∗*p* < 0.001; ∗∗∗∗*p* < 0.0001; ns Not significant, in comparison to the control). HaSGE, *H. anatolicum* salivary gland extract.

A classical hemolysis assay with human, bovine, pig, dog, and chicken serum was performed to understand the effect of HaSGE on the inhibition of complement systems of these hosts. HaSGE inhibited the classical pathway with human and bovine serum in a dose-dependent manner (Fig. 1, *A* and *B*). HaSGE at 1.75 μg and 3.5 μg showed no inhibition of hemolysis with pig serum, while hemolysis inhibition was observed at 7 μg and 14 μg of HaSGE (Fig. 1*C*). Even at 7 μg and 14 μg of HaSGE, only slight hemolysis inhibition was observed for dog and chicken serum (Fig. 1, *D* and *E*). Hemolysis inhibition of different host sera by HaSGE at 1.75 μg was further calculated. HaSGE showed >60% for human and bovine serum, while it inhibited ≤20% for dog serum, negligible for chicken serum, and no inhibition for pig serum (Fig. 1*F*).

**Figure 2:**
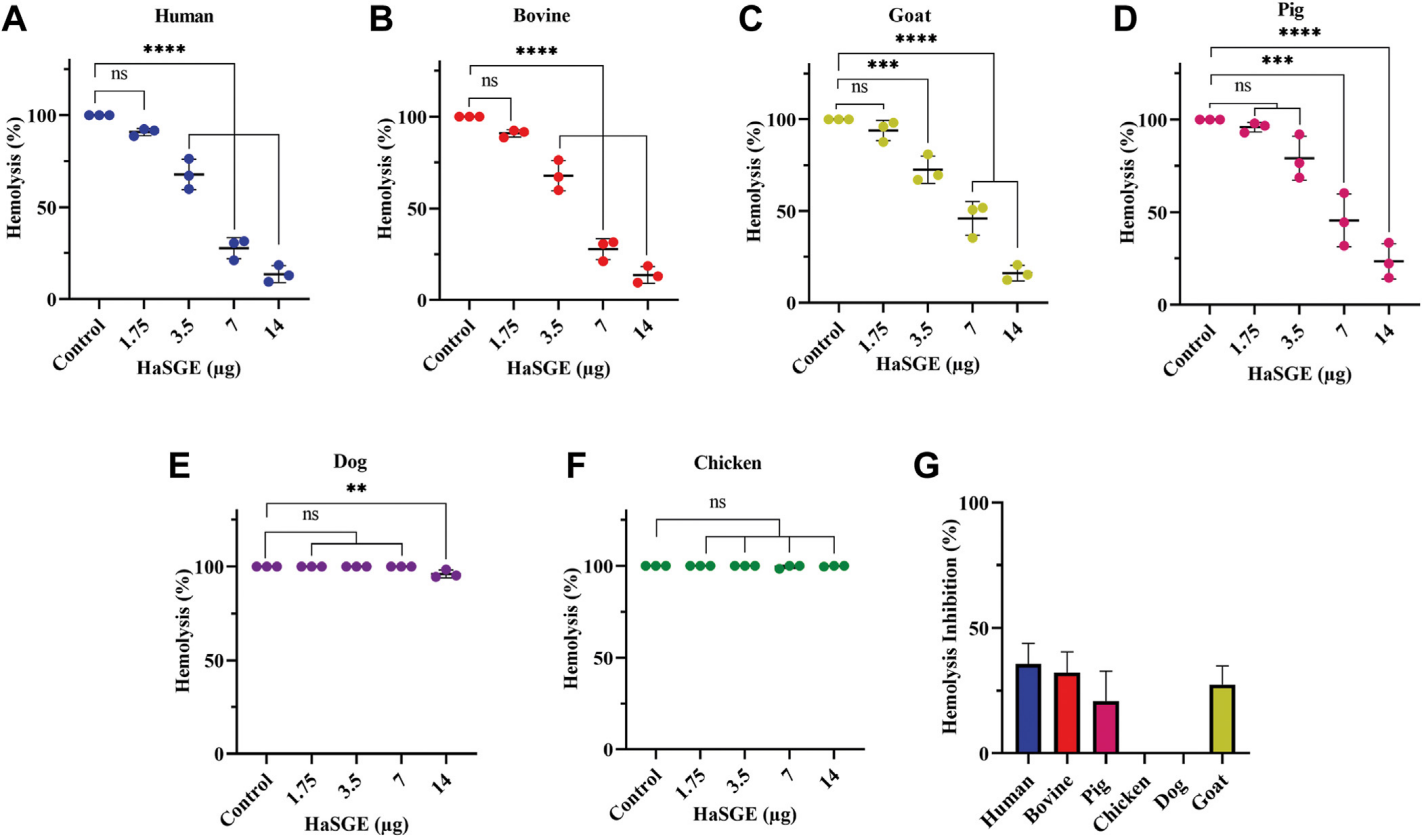
**Effect of HaSGE on the alternative complement pathway using hemolysis assay with different host sera.** Human (*A*), Bovine (*B*), Goat (*C*), Pig (*D*), Dog (*E*), and Chicken (*F*). Serum was preincubated for 30 min at 37 °C with different amounts of HaSGE ranging from 1.75 to 14 μg (in 31 μl). The *X*-axis represents the different amounts of HaSGE ranging from 0 (control) to 14 μg. The *Y*-axis represents the hemolysis in percentage. *G*, hemolysis inhibition at 3.5 μg of HaSGE in different hosts. Each bar represents the hemolysis inhibition of HaSGE on different hosts. The values are expressed as the mean percentage of hemolysis/hemolysis inhibition ± SD from n = 3 independent experiments, each measured in duplicates. Statistical significance was determined using one-way ANOVA (∗∗*p* < 0.05; ∗∗∗*p* < 0.001; ∗∗∗∗*p* < 0.0001; ns Not significant, in comparison to the control). HaSGE, *H. anatolicum* salivary gland extract.

An alternative hemolysis assay was performed using serum from human, bovine, goat, pig, dog, and chicken. The alternative hemolysis assay buffer contains EGTA, which chelates Ca^2+^ ions necessary for activating the classical pathway, thereby selectively allowing the alternative pathway to remain active. HaSGE demonstrated a dose-dependent inhibition of the alternative complement pathway in human, bovine, goat, and pig serum (Fig. 2, *A*–*D*). In contrast, no hemolysis inhibition was observed even at higher amounts (7 μg and 14 μg) of HaSGE for dog and chicken serum (Fig. 2, *E* and *F*). Hemolysis inhibition by 3.5 μg of HaSGE with human, bovine, and goat serum showed ≥25% inhibition. Further, pig serum showed ≤20% inhibition, while dog and chicken serum displayed no inhibition (Fig. 2*G*).

#### HaSGE prevents the deposition of C3 and C5 in the complement pathway

**Figure 3:**
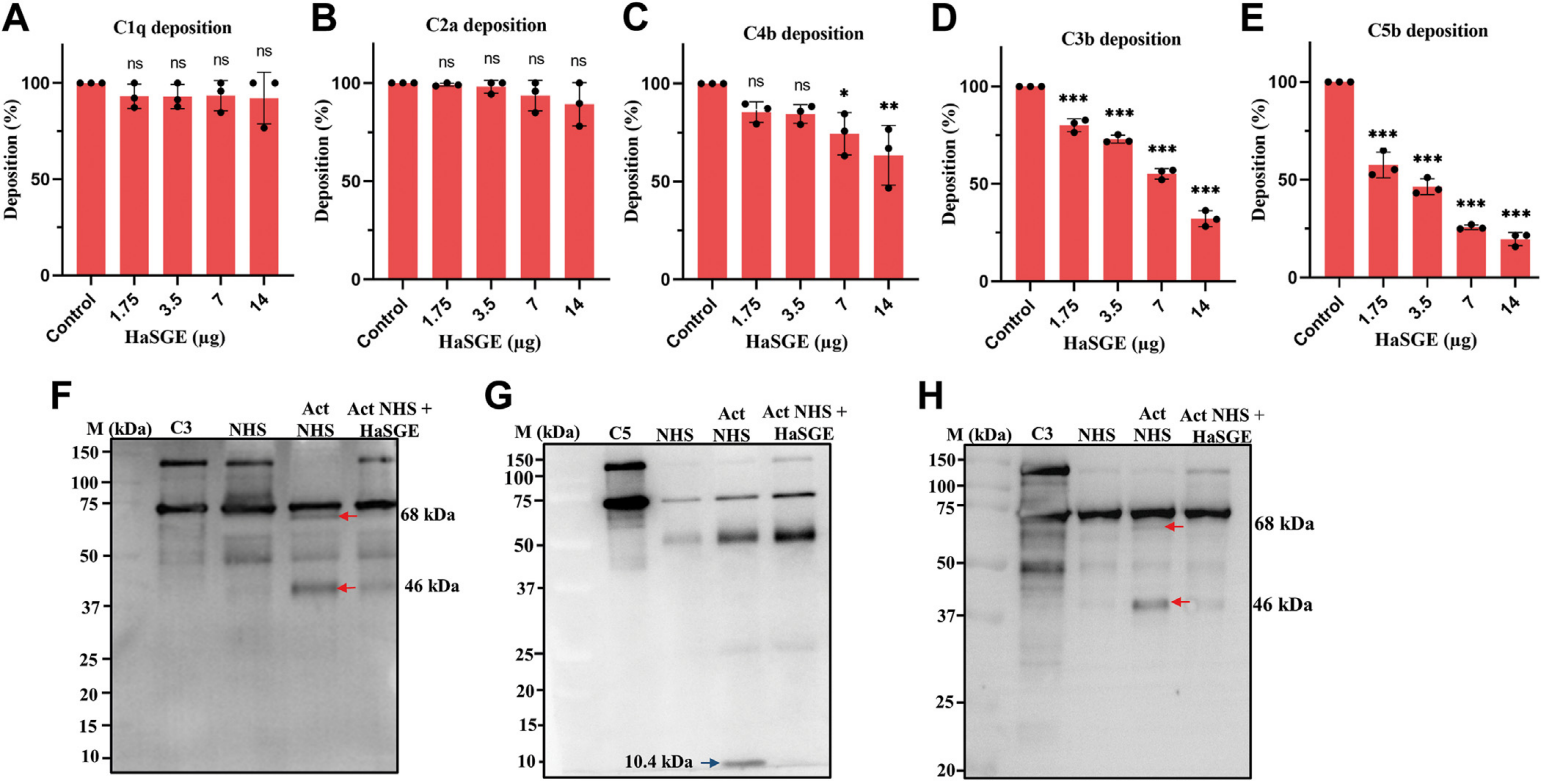
**HaSGE inhibits the classical and alternative complement pathways.** Deposition of C1q (*A*), C2a (*B*), C4b (*C*), C3b (*D*), and C5b (*E*) onto the activation surfaces. The values are expressed as the mean percentage ± SD from n = 3 independent experiments, each measured in duplicates. The *X*-axis represents the different amounts of HaSGE ranging from 0 (control) to 14 μg (in 75 μl). The *Y*-axis represents the deposition rate in percentage. The inhibition of cleavage of the complement proteins C3 and C5 was observed using Western blot analysis. The supernatant from the classical pathway hemolysis assay with or without HaSGE (14 μg) was analyzed by Western blot using anti-C3 (*F*) and anti-C5 (*G*) polyclonal antibodies. The *red arrow* indicates the C3 cleaved fragments (68 kDa) and (46 kDa). The *blue arrow* indicates the C5 cleaved fragment (∼10.4 kDa). *H*. The supernatant from the alternative pathway hemolysis with or without HaSGE (14 μg) was analyzed by Western blot using anti-C3 polyclonal antibodies. *Red arrow* indicates the C3 cleaved fragment (68 kDa) and (46 kDa). M–molecular weight marker, C5, C3-proteins, NHS-normal human serum, Act NHS–supernatant of classical pathway hemolysis without HaSGE. Act NHS + HaSGE-supernatant of classical pathway hemolysis with HaSGE. Purified C3, C5 proteins, and NHS were used as standards in the Western blot analysis. Statistical significance was determined using one-way ANOVA (∗*p* < 0.05; ∗∗*p* < 0.001; ∗∗∗∗*p* < 0.0001; ns Not significant, in comparison to the control). HaSGE, *H. anatolicum* salivary gland extract.

In the deposition assay, surface-bound human immunoglobulin M (IgM) for the classical pathway and mannan for the lectin pathway was used as a complement-activating surface. Normal human serum (NHS) served as the source of complement proteins for these activating surfaces to activate the complement pathway. Upon activation, complement components such as C1q, C2a, C4b, C3b, C5b, and MAC were deposited on the target surface. The effect of HaSGE on the deposition of these complement proteins on activating surfaces was further evaluated using specific antibodies. In the classical pathway, HaSGE did not prevent the accumulation of C1q and C2a on the activating surface (Fig. 3, *A* and *B*). However, the deposition of C4b was impeded at a higher amount of HaSGE, specifically at 7 μg and 14 μg (Fig. 3*C*). Furthermore, the deposition of C3b and C5b was inhibited dose-dependent, with a higher level of significance (Fig. 3, *D* and *E*).

A hemolysis assay was conducted using 14 μg of HaSGE and NHS to elucidate the mechanism of classical pathway inhibition. The supernatant from the reaction mix was subjected to Western blot analysis with anti-C3 and anti-C5 antibodies. Once the complement pathway is activated, C3 is cleaved into C3b and C3a. The C3b fragment, in the presence of the cofactor, is further cleaved by factor I. This results in the formation of inactivated C3b (iC3b) fragments of 68 kDa and 46 kDa (16). We observed 68 kDa and 46 kDa bands only in the activated NHS (Fig. 3*F*). However, in the presence of HaSGE, degraded products (68 kDa and 46 kDa fragments) were absent, suggesting protein(s) present in the HaSGE inhibit cleavage of C3 protein in the NHS (Fig. 3*F*). Similarly, the cleavage of C5 complement protein by C5 convertase at the N terminus of the C5α chain, leading to the release of anaphylatoxin C5a (10.4 kDa), was hindered in the presence of HaSGE (Fig. 3*G*). Protein(s) present in the HaSGE prevents the cleavage of both C3 and C5, thereby avoiding the formation of the MAC.

Similarly, a hemolysis assay was conducted using HaSGE and NHS to elucidate the alternative complement pathway inhibition mechanism. The supernatant from the reaction mix was subjected to Western blot analysis with anti-C3 antibodies. Similar to the classical pathway, 68 kDa and 46 kDa bands indicate degraded products of the C3b. However, these bands were absent in the presence of HaSGE, suggesting protein(s) present in the HaSGE inhibit the cleavage of C3 protein in the NHS (Fig. 3*H*).

**Figure 4:**
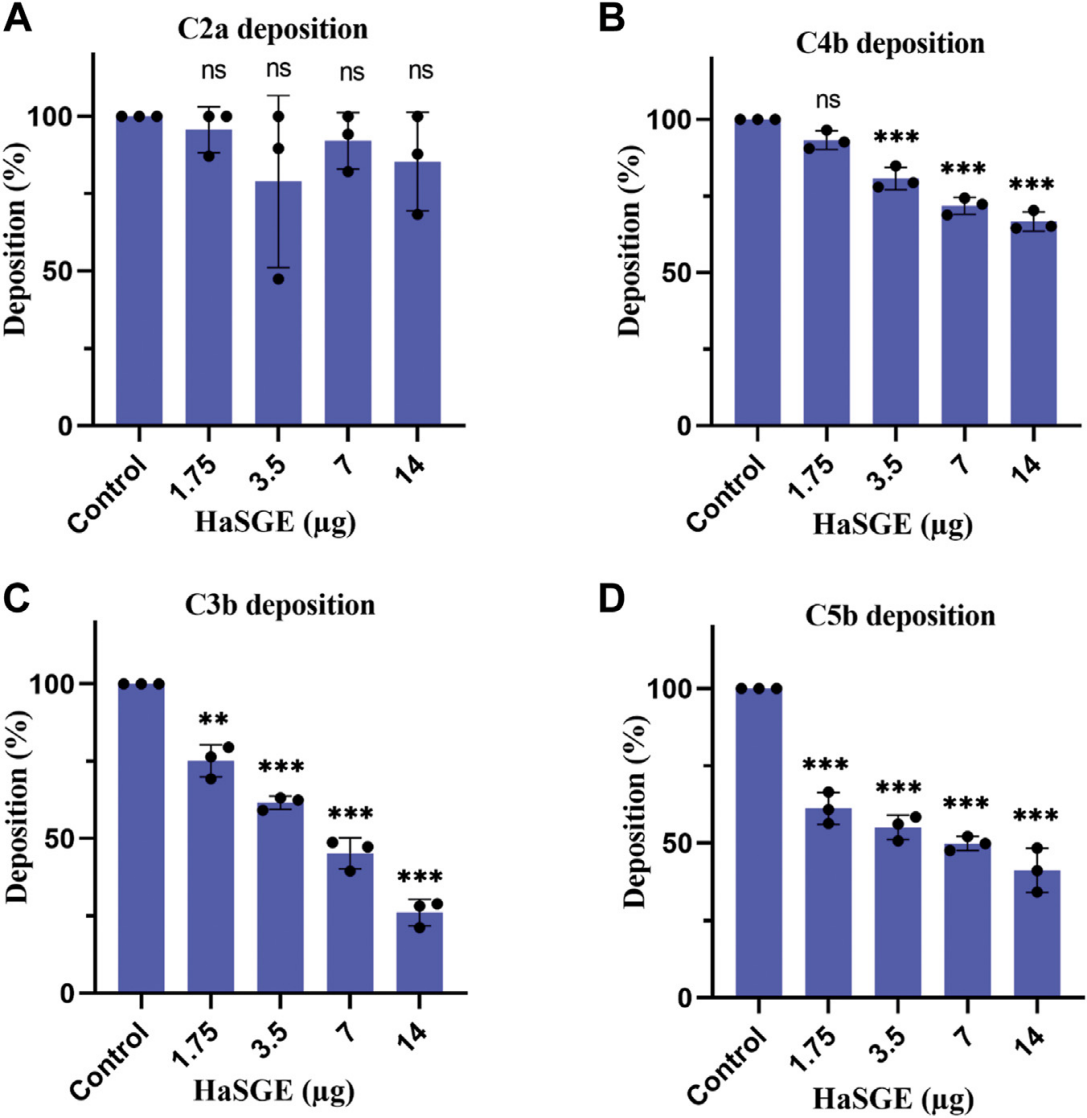
**HaSGE inhibits the lectin complement pathway.** Effect of HaSGE on the deposition of C2a (*A*), C4b (*B*), C3b (*C*), and C5b (*D*) onto the activation surface in the lectin complement pathway. Each bar represents the deposition percentage with different amounts of HaSGE ranging from 0 (control) to 14 μg (in 75 μl). The values are expressed as the mean percentage ± SD from n = 3 independent experiments, each measured in duplicates. Statistical significance was determined using one-way ANOVA (∗∗*p* < 0.001; ∗∗∗∗*p* < 0.0001; ns Not significant, in comparison to the control). HaSGE, *H. anatolicum* salivary gland extract.

Mannan was used as an activating surface in the case of the lectin pathway. Similar to the classical pathway, the deposition of complement proteins on the activating surface was recognized by specific antibodies. HaSGE cannot inhibit the C2a initial component of the cascade, and low inhibition of C4b was observed (Fig. 4, *A* and *B*). However, significant inhibition of C3b and C5b in the presence of HaSGE was observed (Fig. 4, *C* and *D*), which suggests that protein(s) present in the HaSGE also inhibit the lectin complement pathway.

### HaSGE proteins interact with host complement proteins

**Figure 5:**
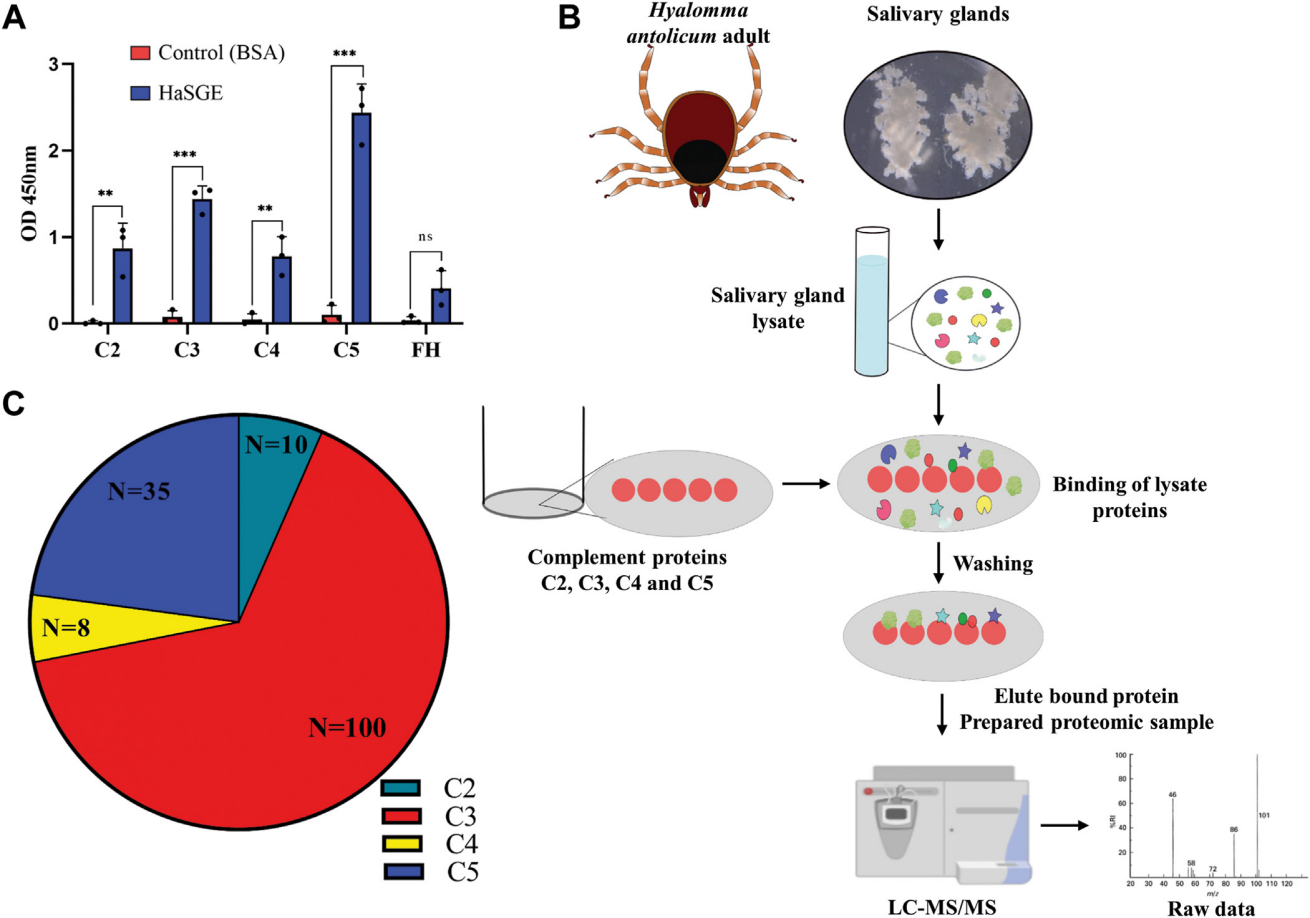
**HaSGE proteins interact with host complement proteins.***A*, interaction of complement component protein with HaSGE. The *Y*-axis represents the absorbance at 450 nm, and the *X*-axis represents the different complements proteins (C2, C3, C4, C5, and FH), probed against their respective antibodies. FH- factor H; BSA-bovine serum albumin, HaSGE- *Hyalomma anatolicum* salivary gland extract. The *bar* represents the mean ± SD from n = 3 independent experiments, each measured in duplicates. Statistical significance was determined using two-way ANOVA (∗∗*p* < 0.001, ∗∗∗*p* < 0.0001). *B*, workflow for identifying HaSGE protein(s) that interact with complement proteins. *C*, the number of proteins in HaSGE that may interact with different complement proteins using LC-MS/MS analysis. BSA, bovine serum albumin; HaSGE, *H. anatolicum* salivary gland extract; LC-MS/MS, liquid chromatography with tandem mass spectrometry.

A direct binding ELISA was performed to identify proteins in HaSGE that can interact with specific host complement proteins. The results suggest that HaSGE interacts strongly with C3 and C5 complement proteins compared to C2 and C4, while no interaction was observed with Factor H (Fig. 5*A*). Bovine serum albumin (BSA) was used as a negative control to monitor the nonspecific binding of complement protein. C5 protein exhibited the strongest interaction with HaSGE among all the tested complement proteins.

Further, a pull-down assay was performed to identify the protein(s) present in the HaSGE that interacts with the host complement proteins. The HaSGE proteins bound to the complement protein were eluted and subjected to liquid chromatography with tandem mass spectrometry (LC-MS/MS) analysis (Fig. 5*B*). Proteins eluted with BSA were used as negative control and removed from the analysis. In-house prepared *H*. *anatolicum* salivary gland long-read transcriptome data (unpublished, submitted BioProject accession number: PRJNA1101950) was used as a reference for the identification of proteins from the LC-MS/MS data. A total of 153 proteins were identified: 10 proteins from C2, 100 proteins from C3, 8 proteins from C4, and 35 proteins from C5 (Fig. 5*C*, Sheet S1).

### HAMpin-1 belongs to the serine protease inhibitor (Serpin) family

**Figure S1:**
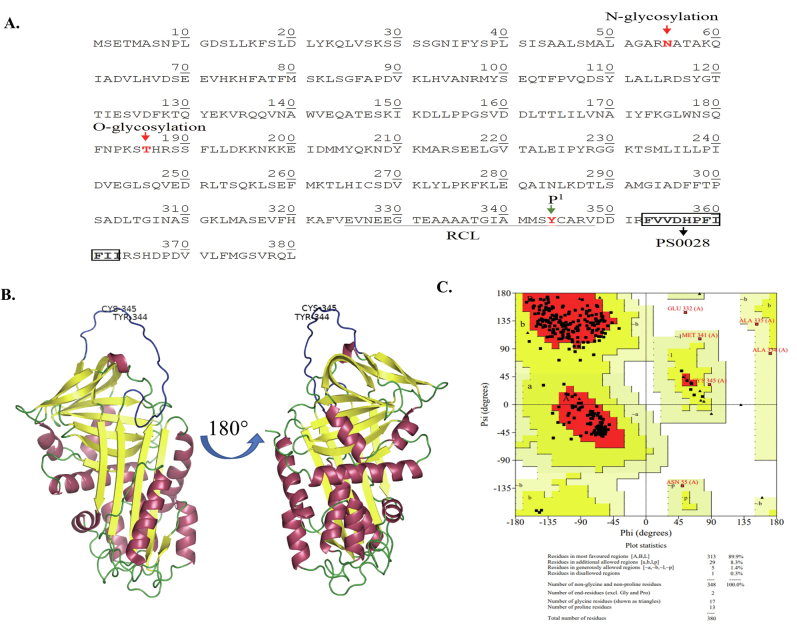
HAMpin-1 sequence and cartoon representation modeled structure. A, The full-length protein sequence of the HAMpin-1. Serpin signature motif PS00284 sequence is shown in the box. The reactive centre loop (RCL) is underlined and the P1 position is shown by the green arrow. Predicted N-glycosylation and O-glycosylation sites are shown in the red arrow. B, Alphafold modelled tertiary structure of HAMpin-1 in front and back view. α-helix: Raspberry red, β-sheet: Pale yellow, RCL: Blue, CYS: Cysteine, TYR: Tyrosine. C, Assessment of HAMpin-1 structure using Ramachandran φ(phi) and ψ(psi) angles with Procheck server.

One serpin family protein was identified in the mass spectrometry (MS) data analysis (Sheet S1). We selected it for further characterization and named it as *Hyalomma anatolicum* serpin-1 (HAMpin-1). The full-length sequence of HAMpin-1 is of 380 amino acid (AA) with a theoretical molecular weight and isoelectric point (pI) of 42 kDa and 5.8, respectively. It contains serpin signature motif PS00284 at 353 to 363 (FVVDHPFIFII) AA positions. It is predicted to have one O-glycosylation site at 186 AA position and one N-glycosylation site at 55 AA (Fig. S1*A*). The modeled structure of HAMpin-1 has a typical serpin tertiary structure consisting of ten alpha helix, three beta sheets, and an RCL protrusion (Fig. S1*B*). Further, this model was validated through the Ramachandran plot, which showed 89.9% of residues (313 AA) fell in the most favored region, 8.3% of residues (29 AA) were in the additional allowed region, 1.4% of residues (5 AA) were in the generously allowed region, and 0.3% of residues (1 AA) in the disallowed region (Fig. S1*C*). The modeled structure of HAMpin-1 was further superimposed on resolved structures of Iripin-1, Iripin-3, Iripin-4, and *Ixodes ricinus* serpin 2 (IRS-2), and the RMSD values 1.281, 1.520, 1.136, and 1.400, respectively (Table S1) were obtained. This confirms that HAMpin-1 shares structural similarities with other tick serpins.

### HAMpin-1 is similar to *Rhipicephalus appendiculatus and Rhipicephalus haemaphysaloides* serpins

**Figure 6:**
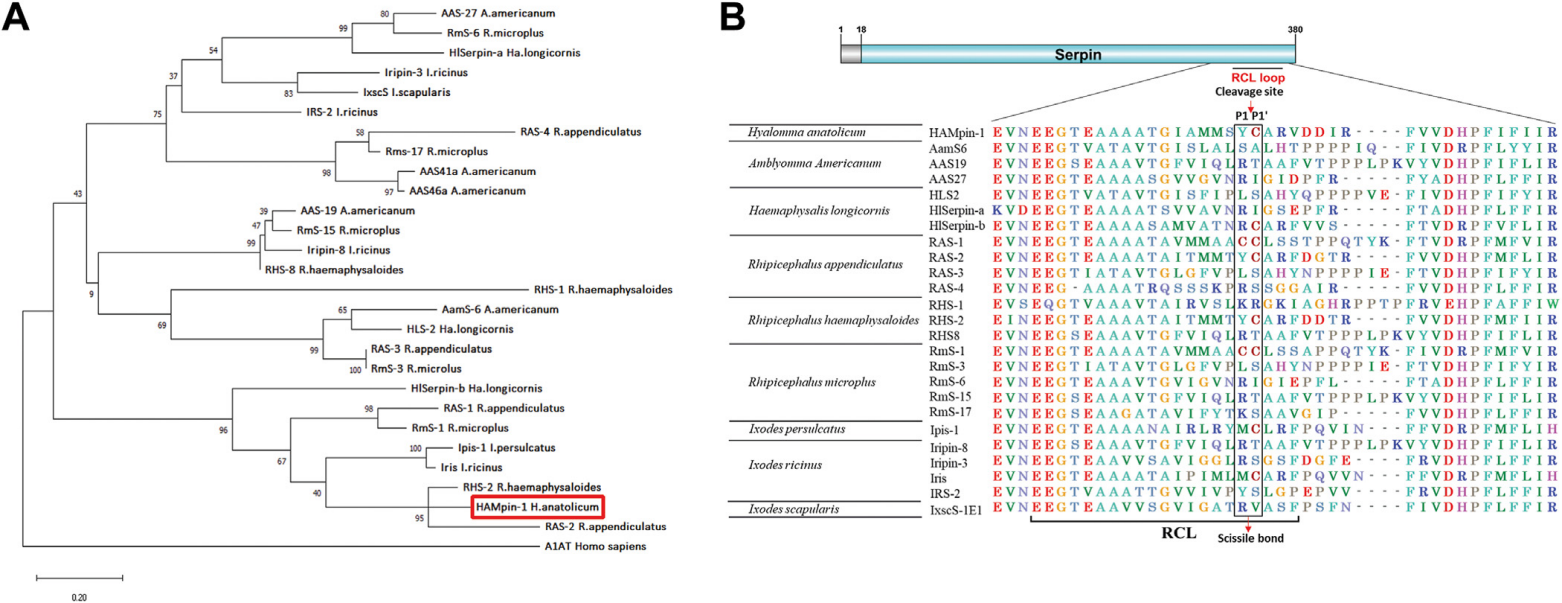
**HAMpin-1 belongs to the serine protease inhibitor (Serpin) family.***A*, the phylogenetic tree was constructed using the protein sequences of functionally characterized tick serpins. The human serpin α-1-antitrypsin (A1AT) was used as an outgroup to root the tree, and a bootstrap value of 1000 replicates was used for clade confidence. *B*, multiple sequence alignment of reactive center loop (RCL) for HAMpin-1 and other tick serpins. P1-P1′ sites are shown in the box. HAMpin-1, *Hyalomma anatolicum* serpin-1.

The amino acid sequence of 27 tick serpins with known function was retrieved from GenBank (Table S2). The phylogenetic analysis of HAMpin-1 with serpins from other tick species shows HAMpin-1 forms a cluster with seven other serpins including HlSerpin-b (*Haemaphysalis longicornis*), RAS-1 (*R. appendiculatus*), RHS-2 (*Rhipicephalus haemaphysaloides*), RmS-1 (*R. microplus*), Ipis-1 (*Ixodes persulcatus*), Iris (*I. ricinus*), and RHS-2 (*R. haemaphysaloides*) (Fig. 6*A*). All these eight serpins, including HAMpin-1 have no signal sequence. HAMpin-1 was closely related to RHS-2 and RAS-2. Multiple sequences alignment of RCL region of HAMpin-1 and other tick serpins shows HAMpin-1 RCL region is partially conserved among different tick species and has tyrosine and cysteine in its P^1^ and P^1^ʹ positions similar to RHS-2 and RAS-2 (Fig. 6*B*).

### HAMpin-1 expression in the tick tissues

**Figure 7:**
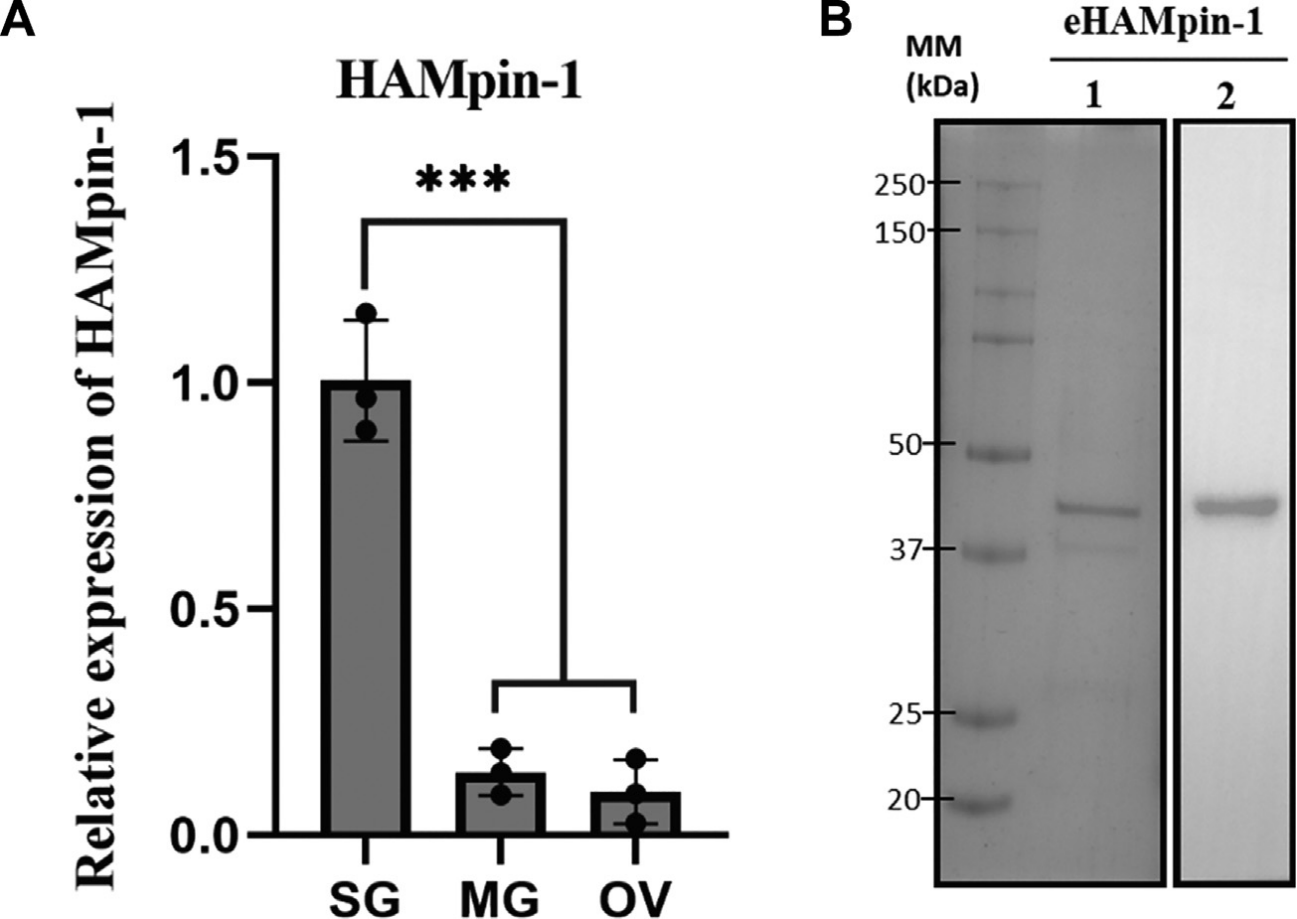
**HAMpin-1 expression in various tissues of***H****yalomma anatolicum* and purified recombinant protein (eHAMpin-1).***A*, RT-qPCR analysis of HAMpin-1 expression in different tissues with reference to *H. anatolicum* actin. Statistical significance was determined using one-way ANOVA (∗∗∗*p* < 0.0001), SG-salivary glands, MG-midgut, OV-ovary. *B*, SDS-PAGE stained with Coomassie brilliant blue dye (1) and Western blot analysis (2) of purified recombinant protein with His tag from prokaryotic expression system (BL21- DE3 *Escherichia coli* cells) probed with HRP conjugated anti-His antibody. eHAMpin-1: *E. coli* expressed *H. anatolicum* serpin-1. HAMpin-1, *Hyalomma anatolicum* serpin-1; RT-qPCR, reverse transcription-quantitative PCR; HRP, horseradish peroxidase.

To assess the expression of HAMpin-1 level in tissues of blood-feeding adult *H. anatolicum* ticks, we conducted reverse transcription-quantitative PCR (RT-qPCR) using complementary DNA (cDNA) prepared from RNA extracted from salivary glands, midgut, and ovaries (Fig. 7*A*). We found that HAMpin-1 is expressed across all the tissues. However, the transcript levels were significantly higher in the salivary glands compared to the midgut and ovaries.

### Recombinant HAMpin-1 is functionally active (serine protease inhibitor) and can inhibit host complement pathway

**Figure 8:**
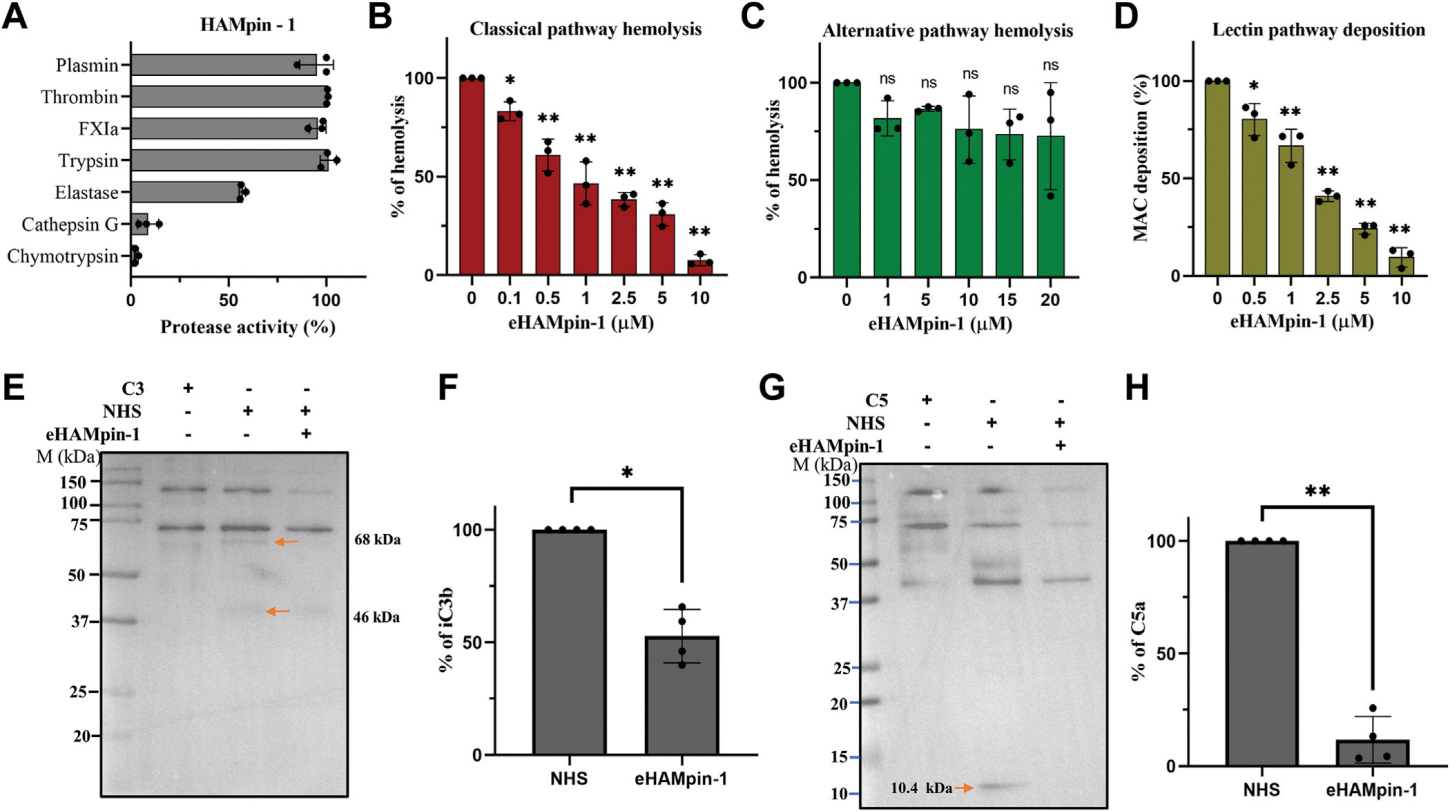
**Functional activity of eHAMpin-1.***A*, the eHAMpin-1 tested for its inhibitory activity on different proteases showed specific protease-inhibitory activity. The *X*-axis represents the percentage of protease activity, and the *Y*-axis represents different tested proteases. The eHAMPin-1 inhibits the classical complement pathway (hemolysis) (*B*), but not the alternative complement pathway (hemolysis) (*C*). The eHAMPin-1 inhibits the MAC deposition in the lectin complement pathway (*D*). The *X*-axis represents the different concentrations (μM) of eHAMpin-1, and the *Y*-axis represents the percentage of hemolysis/deposition. The eHAMPin-1 was found to inhibit the cleavage of C3 (*E*), and C5 (*G*) complements proteins using Western blot analysis. The bar graph represents the quantitative values of C3 (*F*) and C5 (*H*) cleaved products. NHS-normal human serum, eHAMpin-1 - *Escherichia coli* expressed *H.anatolicum* serpin-1. Statistical significance was determined using one-way ANOVA (ns Not significant; ∗*p* < 0.05; ∗∗*p* < 0.0001). HAMpin-1, *Hyalomma anatolicum* serpin-1; MAC, membrane attack complex; NHS, normal human serum.

Serpins are known for their inhibition of serine proteases. We investigated the inhibitory effect of HAMpin-1 on seven serine proteases. The recombinant protein HAMpin-1, was expressed and purified using *Escherichia coli* with His tag (eHAMpin-1). The protein was purified to homogeneity. The eHAMpin-1 resolved at 42 kDa in the SDS PAGE and Western blot analysis (Fig. 7*B*). Further, eHAMpin-1 significantly decreased the proteolytic activity of chymotrypsin and cathepsin-G by more than 90%, while elastase activity was reduced by over 45% (Fig. 8*A*). Notably, the presence of eHAMpin-1 did not affect the proteolytic activity of trypsin, plasmin, thrombin, and factor-XIa (Fig. 8*A*).

Considering the involvement of serine proteases in the complement cascade, we investigated the interference of eHAMpin-1 in all three complement pathways. eHAMpin-1 demonstrated a dose-dependent inhibition of the classical pathway with concentrations ranging from 0.5 to 10 μM (Fig. 8*B*). At a concentration of 1 μM, >50% inhibition of hemolysis was observed, and at 10 μM, inhibition was >90%. However, eHAMpin-1 could not inhibit the alternative complement hemolysis even at higher concentrations of 20 μM (Fig. 8*C*). eHAMpin-1 also inhibited the deposition of MAC on the activating surface in the lectin pathway in dose-dependent concentrations ranging from 0.5 to 10 μM (Fig. 8*D*). Upon confirming the inhibitory activity of eHAMpin-1 in the classical complement pathway, the supernatant of hemolysis was analyzed for cleavage of complement proteins C3 and C5. The HAMpin-1 also inhibited the cleavage of C3 and C5 proteins (Fig. 8, *E*–*H*) suggesting that this protein can inhibit the activation of the host complement pathway during the blood-feeding stage of the tick.

### HAMpin-1 interacts with various host serum proteins

**Figure 9:**
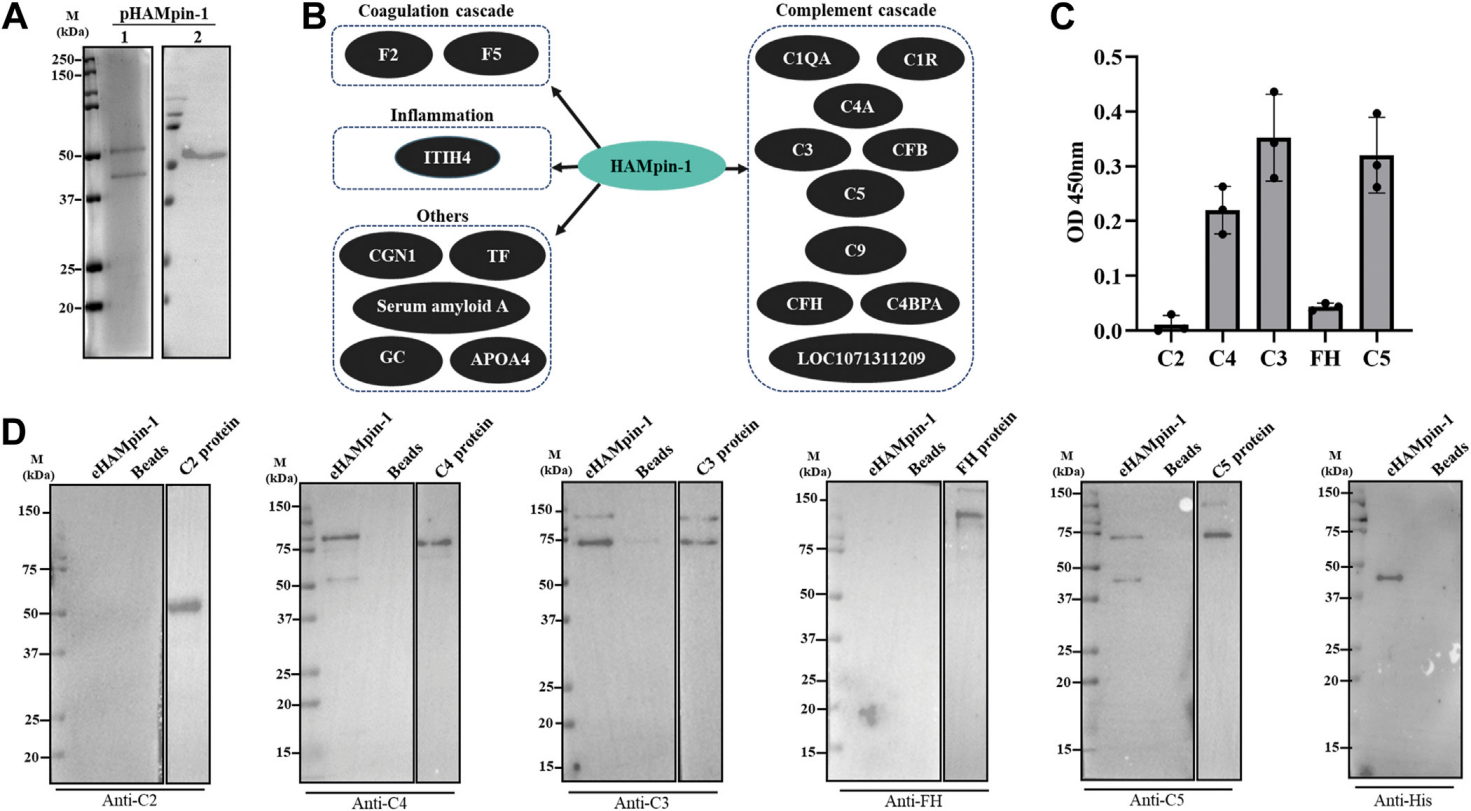
**Proteins interacting with recombinant HAMpin-1.***A*, SDS-PAGE stained with Coomassie brilliant blue dye (1) and Western blot analysis (2) of purified recombinant protein with His tag from the eukaryotic expression system (*Pichia pastoris*). *B*, STRING analysis of the proteins obtained in the NBS (normal bovine serum) pull-down using pHAMpin-1 and after subtraction of proteins obtained from BSA used as a negative control. *C*, ELISA results show the direct interaction of eHAMpin-1 with various complement proteins. The *Y*-axis represents absorbance at 450 nm, and the *X*-axis represents different complement proteins. *D*, Western blot analysis showing the interaction of eHAMpin-1 with complement proteins from NHS (normal human serum). eHAMpin-1 was coated onto beads, or empty beads were used as a control, followed by incubation with NHS. Samples were subjected to Western blot analysis and probed with antibodies against different complement proteins (C2, C4, C3, FH, and C5) and anti-His antibody to detect eHAMpin-1. BSA, bovine serum albumin; HAMpin-1, *Hyalomma anatolicum* serpin-1.

A pull-down assay was conducted using *Pichia pastoris* expressed HAMpin-1 (pHAMpin-1) with bovine serum to investigate the interacting partners of HAMpin-1 in host serum proteins. The recombinant HAMpin-1 protein resolved at 50 kDa in SDS-PAGE analysis, potentially attributed to protein glycosylation (Fig. 9*A*). The pull-down proteins from the bovine serum using pHAMpin-1 as bait were subjected to LC-MS/MS analysis. Twenty-one proteins out of 54 were found to have more than two unique peptides (Sheet S2). Further, complement proteins C3 and C5 were identified with 61 and 24 unique peptides, respectively. Interactome analysis using STRING revealed that HAMpin-1 exhibits interaction with proteins involved in various pathways, grouped in categories such as complement cascade, coagulation cascade, and inflammation (Fig. 9*B*).

The direct interaction of eHAMpin-1 with complement proteins evaluated by ELISA showed eHAMpin-1 could interact with purified complement proteins C4, C3, and C5, whereas no interaction was observed with C2 and FH (Fig. 9*C*). To further validate the direct interaction of eHAMpin-1 with human complement factors under physiological conditions, additional pull-down assays were conducted using NHS, followed by immunodetection of the eHAMpin-1 bound proteins with specific complement antibodies. eHAMpin-1 was able to interact with C4, C3, and C5 complement proteins from NHS, but we did not detect any interaction with C2 and FH (Fig. 9*D*). These results suggest that HAMpin-1 interacts with multiple complement system proteins, thereby modulating host complement pathways.

## Discussion

Ticks, crucial vectors of human and animal disease pathogens, transmit more pathogens than other blood-feeding arthropods, except for mosquitoes (17, 18). *H. anatolicum* is widely distributed, with medical and veterinary significance, posing an economic burden (19, 20). Tick saliva is rich in bioactive molecules, which suppress host defense pathways, leading to successful blood feeding and pathogen transmission (21, 22, 23, 24). Despite the significance of *H. anatolicum* and the wide range of host adaptability (2), there is a notable absence of reports concerning its salivary gland components and their impact on the host complement system.

Tick population dynamics are significantly influenced by host specificity, which dictates the effectiveness of feeding (25, 26). For the first time, we demonstrated the inhibitory effect of HaSGE on all three complement pathways of various hosts. In our study, pronounced inhibition of hemolysis by HaSGE was observed using human, bovine, and goat sera, compared to dog, pig, and chicken sera. Our findings align with existing literature reporting anticomplement activity in saliva or proteins of various tick species, including *I. scapularis, I. ricinus, Ornithodoros moubata, R. microplus, Amblyomma cajennense*, and *Rhipicephalus pulchellus* (5, 6, 11, 13). However, the differential response observed in our study could be due to evolutionary pressure on ticks. This evolutionary pressure makes ticks adapt to a specific host and eventually become their natural host (2, 19). By inhibiting the complement pathways, *H. anatolicum* can evade immediate immune responses, facilitating prolonged feeding. Thus, the interaction between tick salivary proteins and the host's immune system is crucial for the tick's ability to feed, survive, and transmit pathogens in specific hosts.

The evaluation of anticomplement activity through deposition and cleavage assays revealed that HaSGE effectively inhibited the classical and lectin pathways by preventing the C4 deposition in the initial steps, followed by blocking the C3 and C5 cleavage thereby preventing the C3b and C5b deposition. Notably, our findings parallel with previous studies, where the inhibitory effect on the initial stages of the complement pathway by *R. microplus* saliva was observed (5). Studies in *R. microplus* and other hematophagous organisms have shown that calreticulin binds to a C1q protein and prevents the classical complement pathway (27, 28). However, in *A. americanum,* despite calreticulin binding to C1q, it fails to inhibit the classical pathway (29). Notably, in our results, C1q deposition remains unaltered, emphasizing the necessity for further research on calreticulin expression in *H. anatolicum* and its potential interference in the complement pathway. In the presence of HaSGE, a notable inhibition of C3 cleavage was observed, preventing the formation of C3 convertase and the subsequent activation of an alternative pathway. This inhibitory effect resonates with the documented ability of tick molecules such as Isac, Salp2, Irac-1, Irac-2, and CirpA, which have been shown to prevent C3 convertase formation and inhibit the alternative pathway (6, 22). This suggests that the active components within HaSGE act directly on the C3 protein or upstream proteins, effectively arresting the complement pathways.

HaSGE directly interacts with complement proteins C2, C4, C3, and C5, which aligns with the previously reported study on *R. microplus* saliva (5). This observation evidently suggests that there are proteins in the HaSGE that exhibit stronger interactions with specific complement proteins, further leading to the modulation of complement activation and downstream immune responses. MS analysis revealed a set of possible interactors like HSP70, histone, metalloproteases, ZIP10, and serpin. Previous studies demonstrated that HSP70 functions as a chaperone and also protects cells from complement-mediated lysis (30, 31). Histone H3 showed stronger binding to C4b and thereby inhibited the classical and mannose-binding lectin pathways (32). Aureolysin, a metalloprotease from *Staphylococcus aureus*, inhibits the deposition of C3b on bacterial surfaces and the release of the anaphylatoxin C5a (33, 34). ZIP10 protein functions as a cellular regulator to modulate B cell receptor signaling in humoral immune responses (35). The interaction of these *H. anatolicum* salivary gland proteins with the host complement proteins needs to be validated for their different binding and inhibitory ability.

We validated one serpin protein (serine protease inhibitor) from the above. Serpins are one of the four identified serine protease inhibitors family in ticks (36). Serpins use a unique "suicide" mechanism to inhibit their target protease, locking the protease into an irreversible and stable complex and exosites-mediated interactions (37, 38). They regulate various physiological processes, including blood coagulation, angiogenesis, fibrinolysis, inflammation, and complement activation (36, 38, 39). The identified serpin (HAMpin-1) predicted structure exhibited the characteristic features of a typical serpin. The presence of an alanine-rich hinge region (P15-P9 position), and tyrosine at P1 site of the RCL, indicated HAMpin-1 as an inhibitory serpin and would target chymotrypsin-like serine proteases. HAMpin-1 was closely related to *R. haemaphysaloides* (RHS-2) and *R. appendiculatus* (RAS-2). The amino acid tyrosine occupies the P1 position in all these three serpins. HAMpin-1 and RHS2 inhibited the chymotrypsin, while RAS-2 activity has not yet been studied. RHS2 altered the immune response by inhibiting the maturation of bone marrow-derived dendritic cells, and disruption of RHS2 by RNAi led to the decreased tick attachment and engorgement rate (40, 41). On the other hand, the vaccination of cattle with RAS-2 reduced the nymph engorgement rate and increased the mortality rate of female ticks (42). The clade that contains HAMpin-1 has HlSerpin-b (suppress the cytokine production in bone marrow-derived dendritic cells), RAS-1 (anti-tick vaccine candidate), Rms-1 (protease inhibitory activity), IPIS-1 (inhibits proliferation of bovine peripheral blood mononuclear cell and interferon-gamma secretion), and Iris (modulates T lymphocyte and macrophage by inhibiting the production of proinflammatory cytokines) (42, 43, 44, 45, 46). As HAMpin-1 is closely related to these serpins, it might have an immunomodulatory effect on host immune cells, and its potential as a vaccine candidate needs to be further investigated.

HAMpin-1 inhibited the enzymatic activity of chymotrypsin, cathepsin-G, and elastase. As the tick bite induces damage to both the skin and blood vessels, it causes a sharp increase in the levels of α1-antitrypsin and α2-macroglobulin, acting on inhibitors of proteolytic enzymes like trypsin and chymotrypsin at the site of injury (47, 48). If left unchecked, this inhibitor surge could lead to delayed wound healing. Considering HAMpin-1 inhibitory effect on chymotrypsin, it may disrupt the host's tissue repair response at the feeding site and help in continuous blood feeding. Cathepsin G produced by neutrophil cells helps in clearing the engulfed pathogens and regulates inflammation by releasing chemoattractants (49, 50). It also activates proteinase-activated receptors-4, which induce proinflammatory signals, pain, and platelet aggregation (48). Elastase is released by neutrophils and stored in azurophil granules and involved in antimicrobial defense and destroys pathogens, and it also plays a role in inflammatory regulations (51, 52). HAMpin-1 mediated inhibition of these proteases facilitates continuous *H. anatolicum* feeding by suppressing the immune response and delaying wound healing.

Hematophagous ectoparasites take whole blood, which reaches their gut. These ectoparasites must develop methods to protect themselves from complement damage. Research on scabies mite serpins like SMSB3 and SMSB4 demonstrated complement system inhibition, thereby safeguarding the mite digestive system from complement-mediated lysis (53). *I. ricinus* tick escapes from the alternative complement pathway of the host by secreting serpins, Iripin5, and Iripin8, from the salivary glands (11, 54). Our results demonstrate that HaSGE inhibits all three host complement pathways, whereas HAMpin-1 specifically modulates the classical and lectin pathways. Previous studies have also shown that the components of tick saliva exhibit moonlighting protein property (a single protein can target multiple functions) as well as redundancy property (many proteins share overlapping functions) in modulating host immune response (55). The molecule(s) in HaSGE, which modulates the host alternative pathway, requires further investigation.

Expression of HAMpin-1 in salivary glands, midgut, and ovaries suggests that it plays various physiological functions. A higher transcript level was observed in salivary glands. Notably, previous studies showed that serpin RHS8 from *R. haemaphysaloides* plays a role in reproduction; knockdown caused a reduction in vitellogenin proteins and impaired oocyte maturation (56). The presence of HAMpin-1 transcript in ovaries suggests that it might play a role in the reproductive process, which needs to be investigated. Further, the presence of HAMpin-1 transcript in the midgut suggests that HAMpin-1 might play a protective role in the gut by shielding it from complement-mediated attack like scabies mites (53).

HAMpin-1 also interacts with various host serum proteins, as evidenced by the identification of several proteins in pull-down analysis. It showed the interaction with complement cascade proteins and coagulation cascade proteins factor II (prothrombin) and factor V (proaccelerin) important molecules in a common coagulation cascade. Activated factor Va interacts with factor Xa to form a prothrombinase complex, which catalyzes prothrombin to thrombin. Thrombin catalyzes the conversion of fibrinogen to fibrin, which forms a stable blood clot (57, 58). The interaction of HAMpin-1 with prothrombin, a serine protease, the central protein in the coagulation cascade, and proaccelerin needs to be further investigated. The interactome of HAMpin-1 showed interaction with interalpha-trypsin heavy chain H4 (ITIH4), an inflammatory regulator (59), transporter proteins like apolipoprotein, serotransferrin, primary-amine oxidase, and uncharacterized protein. Further, exploring these interactions may provide insights into the cross-talk between these pathways and its implications for the pathophysiology of related infections. The serpins can form a stable complex with proteases and can interact with complement proteins (53). Since HAMpin-1 does not form a stable complex with the complement proteins as it forms with chymotrypsin (data not shown), further studies are required to understand the interaction of HAMpin-1 with these nonprotease factors.

In conclusion, our results provide additional support for the evasion of host complement proteins by the ticks. We demonstrated that HaSGE can impede the complement cascade in a host-specific manner by directly interacting with different complement proteins. Furthermore, we identified and characterized HAMpin-1, which possesses protease inhibitory function and interacts with various components in host blood serum but primarily with complement proteins. Finally, we validated its role in inhibiting the activation of the host complement pathway (classical and lectin pathways) through direct interaction with several complement proteins.

## Experimental procedures

### Ethical clearance

The present study followed national and institutional guidelines for animal treatment. Ethical approval for this study was obtained from the Institutional Animal Ethical Committee (IAEC) with an approval number: IAEC/NIAB/2023/28/AS.

### Tick and salivary gland lysate

*H. anatolicum* ticks were collected from their natural bovine host. The ticks were sterilized using 70% ethanol for 4 to 5 min, followed by washing with PBS. Subsequently, the ticks were placed on double-sided tape, dissected from a dorsal view, and the salivary glands were isolated. The isolated glands were further washed with PBS and stored in specific assay buffers based on the intended downstream analysis: alternative pathway buffer (20 mM Hepes, 145 mM NaCl, 5 mM EGTA, 7 mM MgCl_2_, and 0.1% gelatin, pH 7.4), classical pathway (CP) buffer (20 mM Hepes, 145 mM NaCl, 5 mM MgCl_2_, 2.5 mM CaCl_2_, and 0.1% gelatin, pH 7.4), HEPES-NaCl-MgCl₂-EGTA buffer (HNEM buffer) (20 mM Hepes, 144 mM NaCl, 5 mM EGTA, and 7 mM MgCl_2_, pH 7.4), or HEPES-NaCl-MgCl₂-CaCl₂ buffer (HNMC buffer) (20 mM Hepes, 144 mM NaCl, 2.5 mM MgCl_2_, and 2 mM CaCl_2_, pH 7.4).

To extract the contents of the salivary glands, they were subjected to sonication for 1 min using cycles of 5 s ON and 10 s OFF, with an amplitude of 30%. The resulting cell lysate was centrifuged at 4 °C for 25 min at 20,000*g*. The supernatant was carefully collected and stored at −80 °C for future use. The protein concentration in the HaSGE was quantified using the Pierce bicinchoninic acid assay (BCA) protein assay kit (Cat no: 23225) for accurate measurement.

### Serum source and erythrocytes

Blood samples were obtained from various species, including cows (*Bos indicus*), dogs (*Canis familiaris*), chickens (*Gallus gallus*), goats (*Capra hircus*), and pigs (*Sus scrofa*). The collected samples were allowed to undergo clotting at 37 °C for 30 min. Subsequently, the samples were centrifuged at 5000*g* for 5 min at 4 °C. Sera from respective species were pooled together and stored at −80 °C for future use. On the other hand, for isolation of erythrocytes, blood samples from sheep (*Ovis aries*) and rabbits (*Oryctolagus cuniculus*) were collected using tubes containing acid-citrate-dextrose (ACD) solution. For every 1.5 volumes of ACD solution, 10 volumes of blood were conserved. NHS was purchased from Sigma-Aldrich (Cat no. S1-100ML).

### Classical complement pathway hemolysis assay

The impact of HaSGE on the classical complement pathway was investigated using a modified hemolytic assay based on the method described previously (60). Sheep erythrocytes preserved in ACD solution were washed thrice with CP buffer through centrifugation at 480*g* for 3 min at 4 °C. The cells were then suspended in the same CP buffer and sensitized by an anti-sheep red blood stroma antibody (diluted 1:100 in CP buffer). The sensitized erythrocytes were incubated at 37 °C for 30 min under gentle agitation. Following two additional washes with CP buffer, the final cell concentration was adjusted to 1 × 10^7^ cells/ml.

For the assay, a mixture of 25 μl sensitized sheep erythrocytes, 25 μl diluted serum (with dilution ratios of 1:10 for NHS, 1:5 for cow serum, 1:3 for dog serum, 1:5 for pig serum, and 1:10 for chicken serum), and 6 μl of the desired concentration of HaSGE was prepared and incubated at 37 °C for 30 min with gentle shaking (600 rpm). Sera dilution was optimized for different hosts to achieve a concentration sufficient to lyse approximately 90% of the erythrocytes. Subsequently, the tubes were centrifuged at 480*g* for 3 min at 4 °C. Following centrifugation, 25 μl of the supernatant (corresponding to the lysed erythrocytes) was transferred to a 96-well microplate containing 70 μl of autoclaved MilliQ water. The extent of hemolysis was measured in a microplate reader at 414 nm. Each experiment was performed in duplicate with three biological replicates. Two control samples were included: a negative control where sera were substituted with an equal volume of CP buffer and a positive control where sera were added without HaSGE. The results were expressed as a percentage of hemolysis using the following formula:

% of hemolysis = [(sample *A*_414_ - negative control *A*_414_)/(positive control *A*_414_ - negative control *A*_414_)] ∗ 100.

### Alternative complement pathway hemolysis assay

A modified hemolysis assay was performed to examine the effect of HaSGE on the alternative complement pathway as described previously (60). Rabbit blood preserved in ACD solution was used for the experiments. Rabbit erythrocytes were washed thrice with alternative pathway buffer through centrifugation at 480*g* for 3 min at 4 °C. Subsequently, the final cell concentration was adjusted to 1 × 10^7^ cells/ml and used as activating surfaces for the alternative complement pathway.

For the assay, sera from different hosts, namely human, cow, goat, chicken, dog, and pig were used at varying dilutions of 1:10, 1:5, 1:13, 1:10, 1:3, and 1:3, respectively. The assay followed a procedure similar to the classical pathway but using alternative pathway-specific buffers.

### Deposition assay for classical and lectin pathway

The classical pathway analysis was performed on 96-well ELISA plates. First, the plates were sensitized with purified human IgM at a concentration of 5 μg/ml in 75 μl of bicarbonate/carbonate coating buffer (35 mM NaHCO_3_, 15 mM Na_2_CO_3_, pH 9.6) and incubated overnight at 4 °C. After sensitization, the wells were washed with washing solution (10 mM Tris, 140 mM NaCl, 5 mM CaCl_2_, 0.1% skimmed milk powder, pH 7.4). The wells were blocked with solution A (10 mM Tris, 140 mM NaCl, and 3% skimmed milk powder, pH 7.4) and then with solution B (10 mM Tris, 140 mM NaCl, 5 mM CaCl_2_, and 3% skimmed milk powder, pH 7.4), each for 30 min at room temperature (RT).

For the assay, diluted NHS (1:20) in HNMC buffer and HaSGE at the desired concentration were added to the wells with a final volume of 75 μl in sample buffer. After incubation at 37 °C for 30 min, the wells were washed with the washing solution. The components deposited on the plate surface were detected by adding 75 μl/well of specific antibodies like goat anti-human C1q (CompTech: A200) at a 1:10,000, goat anti-human C2 (CompTech: A212) at a 1:10,000, goat anti-human C4 (CompTech: A205) at a 1:50,000, goat anti-human C3 (CompTech: A213) at a 1:50,000, and goat anti-human C5 (CompTech: A220) at a 1:5000. All the antibodies were diluted in HN buffer (10 mM Hepes, 140 mM NaCl, pH 7.4) and incubating for 30 min at RT. Following this, wells were washed and incubated with peroxidase-conjugated anti-goat IgG antibody at a 1:2000 dilution in HN buffer for 30 min at RT. The bound components were detected using tetramethyl benzidine/hydrogen peroxide solution. The reaction was stopped by 2M sulfuric acid. The absorbance was measured at 450 nm using an ELISA plate reader. Each set of experiments included two controls: a positive control (NHS without HaSGE protein) and a negative control (only buffer). The positive controls were considered as 100% deposition.

In the lectin pathway, 96-well ELISA plates were coated with 20 μg/ml of mannan from *Saccharomyces cerevisiae* (Sigma-Aldrich-M7504) in bicarbonate/carbonate coating buffer, followed by an overnight incubation at 4 °C. The wells were washed with Tris buffered saline containing 0.01% Tween 20 (TBST) followed by blocking with 3% BSA for 2 h at RT under agitation. Further, NHS (1:20) was added to HEPES-NaCl-MgCl₂-CaCl₂ (HNMC) buffer and the desired concentration of HaSGE/HAMpin-1 in a final volume of 75 μl in sample buffer and incubated at 37 °C for 30 min. The components deposited on the plate surface were detected by adding 75 μl/well of specific antibodies like goat anti-human C2 (CompTech: A212) at a 1:10,000, goat anti-human C4 (CompTech: A205) at a 1:50,000, goat anti-human C3 (CompTech: A213) at a 1:50,000, and goat anti-human C5 (CompTech: A220) at a 1:5000 and MAC C5b-9 antibody (Santa Cruz Biotechnology) at 1:3000. All the antibodies were diluted in 1% BSA. After incubating for 2 h with specific antibodies, wells were washed and incubated with peroxidase-conjugated anti-goat IgG antibody at a 1:2000 dilution in 1% BSA and further incubated for 2 h at RT. The bound components were detected as mentioned above.

### Western blot to identify cleavage of complement proteins C3 and C5

The effect of HaSGE on the cleavage of C3 and C5 complement proteins in NHS was analyzed using Western blotting. The supernatants obtained from the classical and alternative pathway hemolytic assays with or without HaSGE at 14 μg were electrophoresed using a 12% SDS-PAGE under reducing conditions and transferred onto the polyvinylidene fluoride membrane. Specific antibodies were used for goat anti-human C3 (CompTech: A213) at 1:10,000 and goat anti-human C5 (CompTech: A220) at 1:5000 dilutions. After incubation with rabbit anti-goat IgG peroxidase-conjugated secondary antibody and developed using femtoLUCENT PLUS-horseradish peroxidase (HRP) substrate kit following manufactures protocol.

### ELISA for identification of complement components directly binding to HaSGE

The 96-well microplates were coated with 10 μg of HaSGE protein and 1% BSA (negative control) in 50 μl of bicarbonate/carbonate buffer and incubated overnight at 4 °C. Subsequently, the wells were washed with phosphate-buffered saline with 0.01% Tween 20 (PBST) solution and blocked with 1% BSA in PBS for 1 h at RT to prevent nonspecific binding. Following this, wells were incubated with 0.2 μg of complement proteins (C2, C3, C4, C5, and factor H) diluted in PBS to a final volume of 50 μl under agitation at 37 °C for 30 min. The bound complement proteins were detected by incubating with specific antibodies: goat anti-human C2, goat anti-human C3, goat anti-human C4, goat anti-human C5, and goat anti-human factor H, followed by washing, incubation with peroxidase-conjugated anti-goat IgG secondary antibody and detection using tetramethyl benzidine/hydrogen peroxide solution. The reaction was stopped by 2M sulfuric acid. The absorbance was measured at 450 nm using an ELISA plate reader.

### Pull-down assay

The 96-well plates were coated with 0.2 μg of C2, C3, C4, and C5 complement proteins, along with 1% BSA (used as a negative control), in bicarbonate/carbonate coating buffer and incubated overnight at 4 °C. After this, the wells were washed, blocked with 1% BSA, and incubated with 10 μg of HaSGE for 30 min at 37 °C under agitation. The wells were washed, bound proteins were eluted with 0.2 M glycine, pH 2.3, and neutralized with 1M Tris–HCl, pH 9.

### MS analysis of pull-down protein samples

The eluted protein samples from pull-down were subjected to reduction by adding 20 mM DTT and incubated at 57 °C for 1 h with shaking. After reduction, the samples underwent alkylation by adding 20 mM iodoacetamide and incubated for 1 h at RT in the dark. Next, trypsin was added to the samples at a ratio of 1:30 (trypsin: protein) and incubated overnight at 37 °C for digestion, followed by adding 0.1% trifluoroacetic acid to arrest the digestion reaction. The digested peptides were purified using C18 spin columns and subjected to vacuum evaporation to remove excess solvents and concentrate the peptide samples. Finally, the peptide samples were reconstituted with 0.1% formic acid.

LC-MS/MS was performed using a Q Exactive HF-Orbitrap mass spectrometer (Thermo Fisher Scientific) with an Ultimate 3000 RSLCnano LC system (Thermo Fisher Scientific). Column temperature and flow rate were set to 20 °C and 0.200 μl/min, respectively. Five microliters of the sample was injected, and separated through a reverse-phase C18 column (PepMap RSLC C18, 2 μm, 100 Å, 75 μm × 50 cm; Thermo Fisher Scientific) by a gradient flow of solvent B (0.1% formic acid in 80/20 acetonitrile/water) from 5%, 40%, 70%, and 90% in 180 min. A full MS scan was performed with mass to charge (m/z) of 375 to 1600 with a resolution of 60,000. The top 25 intense peaks were fragmented in MS/MS with a resolution of 15,000 with a fixed first mass of 100 m/z. Thermo Proteome Discoverer Version 2.4 (https://thermo.flexnetoperations.com/control/thmo/login) was used to examine the obtained spectra. The database search was carried out using SEQUEST HT with the default parameters: full trypsin digestion specificity, maximum missed cleavage sites of 2, fragment mass tolerance of 0.02 Da, and precursor mass tolerance of 10 ppm. Fixed modification, carbamidomethylation of Cys (cysteine), dynamic modification, and oxidation of Met (methionine) were added. The resulting peptides were validated using a fixed value peptide–spectrum match validator with default Maximum Delta Cn and Maximum Rank values. In this analysis, we used a reference file of the predicted proteins from the in-house prepared *H*. *anatolicum* salivary gland long-read transcriptome data (unpublished, submitted BioProject accession number: PRJNA1101950) to identify the proteins in the pull-down samples.

### Bioinformatics analysis

The molecular weight and isoelectric point of HAMpin-1 were computed using Compute pI/Mw tool. Serpin signature motif PS00284 was identified using the ScanProsite tool (61). NetOGlyc 4.0 and NetNGlyc 1.0 were used to predict O-glycosylation and N-glycosylation sites, respectively (62, 63). Phylogenetic analysis of HAMpin-1 with other functionally characterized tick serpins including AamS6, AAS19, AAS27, and AAS41 from *A. americanum* (64, 65); Ipis-1 from *I. persulcatus* (45); HLS2, HlSerpin-a and HlSerpin-b from *Hyalomma longicorni* (43, 66); Iripin-8, Iripin-3, Iris, and IRS-2 from *I. ricinus* (11, 46, 67, 68); IxscS-1E1 from *I. scapularis* (69); RAS-1, RAS-2, RAS-3, and RAS-4 from *R. appendiculatus* (70); RHS-1, RHS-2, and RHS8 from *R*. *haemaphysaloides* (40, 56); and RmS-1, RmS-3, RmS-6, RmS-15, and RmS-17 from *R. microplus* (44) was carried out using the Maximum Likelihood method with Jones Taylor Thornton (JTT) model for 1000 bootstrap replicates using MEGA 10 (https://www.megasoftware.net). Human alpha-1-antitrypsin (A1AT) (71) was used as an outgroup. Multiple sequence alignment of the RCL region was carried out using MUSCLE algorithm.

HAMpin-1 structure was modeled using AlphaFold (https://alphafold.ebi.ac.uk/), and the structure was validated by Ramachandran plot using SAVESv6.0 web server. X-ray crystallography structures of Iripin-1 (72), Iripin-3 (67), Iripin-4 (73), and IRS-2 (74) were obtained from the Protein Data Bank database. Using the PyMOL tool, these structures were visualized and superimposed on the predicted tertiary structure of HAMpin-1. The respective RMSD values were then calculated.

### Cloning, expression, and purification of HAMpin-1

RNA was extracted from the salivary gland of *H. anatolicum* ticks using an MN extraction kit (Takara). A total of 1 μg RNA was taken as a template for cDNA synthesis using the PrimeScript 1st strand cDNA Synthesis Kit (Takara). The target gene sequence encoding the *Hyalomma* Serpin (HAMpin-1) (unpublished, submitted BioProject accession number: PRJNA1101950) was amplified from the cDNA template utilizing primers listed in Table S3. The HAMpin-1 gene was cloned into the pET21a vector inframe with the C-terminal His tag, using Not1 and Nde1 restriction sites. The transformed *E. coli* BL21 (DE3) cells were induced for protein expression using 200 μM IPTG at 18 °C for 16 h. Following incubation, cells were pelleted at 8000*g* for 10 min and lysed using Tris-NaCl buffer, followed by sonication. *E.coli* expressed HAMpin-1(eHAMpin-1) was purified in native conditions using Ni-NTA agarose affinity chromatography and dialyzed in 20 mM Tris–HCl, NaCl 150 mM pH 7.4. Protein concentration was determined by BCA. The recombinant HAMpin-1 (eHAMpin-1) was used for the hemolysis assay for classical and alternative complement pathways using the method described above. The ImageJ software (https://imagej.net/ij/download.html) was used for quantification of the blot.

For yeast expression, HAMpin-1 open reading frame was cloned into pPICZα-A vector in frame with α-factor and C-terminal His-tag using KpnI and XbaI sites, primers listed in Table S3. The pPICZα-A/HAMpin-1 expression plasmid was linearized with PmeI restriction enzyme and electroporated into *P. pastoris* X-33 strain according to the manufacturer's recommendations (Invitrogen, Life Technologies). Transformed colonies were selected on yeast extract peptone dextrose medium agar plates containing zeocin (100 μg/ml) and incubated at 28 °C. After 3 days, colonies were screened with gene-specific primers and vector-specific primers by colony PCR (Table S3). Positive colonies were inoculated in buffered glycerol complex medium and grown overnight at 28 °C with shaking (240 rpm). Subsequently, cells were collected and inoculated in buffered methanol-complex medium to the final *A*_600_ of 1.0 and grown at 240 rpm at 28 °C for 3 days. Protein expression was induced every 24 h by adding methanol to 0.5% final concentration. Recombinant proteins in culture media were precipitated by ammonium sulfate at 75% saturation (516 g/L of media) with stirring at 4 °C overnight. The precipitate was pelleted at 15,000*g* for 1 h at 4 °C. The pellet was resuspended and dialyzed in buffer containing 20 mM Tris–HCl, 500 mM NaCl, pH 7.4. *P. pastoris* expressed HAMpin-1 (pHAMpin-1) was confirmed by resolving samples on a 12.5% SDS-PAGE and Western blotting analysis using Penta-His HRP conjugate antibody (1:5000 dilution) (Cat no: 34460, Qiagen). A positive signal was detected using a femtoLUCENT PLUS-HRP substrate Kit. Affinity-purified protein was dialyzed using 20 mM Tris–HCl, NaCl 150 mM buffer pH 7.4. Protein concentration was determined by BCA and stored at −80 °C until use.

### Proteases inhibition assay

The inhibitory activity of eHAMpin-1 against a set of seven selected mammalian host serine proteases was tested, following the method described previously (68). Serine proteases tested are α-chymotrypsin (96 nM) from bovine pancreas (cat no. C4129-5 mg, Sigma-Aldrich), trypsin (0.3 nM) from bovine pancreas (cat no. T8003-100 mg, Sigma-Aldrich), elastase (100 nM) from porcine pancreas (cat no. E250-10 mg, Sigma-Aldrich), factor XIa (3.68 nM) from bovine (cat no. BFXIA 85L, Enzyme Research), plasmin (15 nM) from human (cat no. HPlas2500PA-1 mg, Enzyme Research), thrombin (250 nM) from bovine plasma (cat no. T7326-1ku, Sigma-Aldrich) and Cathepsin G (110 nM) from human leukocytes (cat no. 4428, Sigma-Aldrich). Assay buffer for chymotrypsin, trypsin, and plasmin: 50 mM Tris, 150 mM NaCl, 20 mM CaCl_2_, 0.01% Triton X-100, pH 8. For factor XIa, cathepsin-G, and thrombin: 20 mM Tris, 150 mM NaCl, 0.1% BSA, pH 7.4 and for elastase: Hepes 50 mM, 100 mM NaCl, Triton X-100, pH 7.4, were prepared. Substrates at a concentration of 0.25 mM were used. The substrate included Succinyl-Ala-Ala-Pro-Phe-p-nitroanilide (cat no. 14993) for cathepsin-G and α-chymotrypsin, D-Val-Leu-Lys-p-nitroanilide (cat no. 36580) for plasmin, Suc-Ala-Ala-Ala-pNA (cat no. 36478) for elastase, D-Pro-Phe-Arg-pNA (hydrochloride) (cat no. 27569) for factor XIa were purchased from Cayman, and Bz-Phe-Val-Arg-pNA (cat no. 920-13) for trypsin and thrombin was purchased from Echelon Biosciences. Subsequently, 1 μM eHAMpin-1 was preincubated with optimized amount of protease enzymes for 30 min at 37 ˚C in the indicated assay buffer. A total of 0.25 mM corresponding substrate was added to the reaction mix in 100 μl final reaction volume in 96 well plates. Substrate hydrolysis was measured at 405 nm every 15 s for 30 min using a multimode plate reader. The enzyme activity level was determined using the formula: (V_i_/V_e_) ∗100, where V_i_ = enzyme activity in the presence of HAMpin-1, V_e_ = enzyme activity in the absence of HAMpin-1. Data were presented as mean ± standard deviation of three independent replicates performed in duplicates.

### HAMpin-1 expression using RT-qPCR

Different tissue samples, including salivary glands, midgut, and ovaries, were isolated from adult *H. anatolicum* ticks. RNA extraction and cDNA conversion were performed as described above. RT-qPCR was performed using fivefold diluted cDNA mixed with SYBR Premix Ex Taq master mix (Cat no. RR420S, Takara) and gene-specific primers in CFX96 real-time PCR system (Bio-Rad). Cycling conditions for HAMpin-1 were 95 °C for 3 min followed by 36 cycles of 95 °C for 10 s, 55 °C for 30 s. The relative quantification of HAMpin-1 in different tissue samples was performed using 2^-▵▵CT^ method (75). *H*. *anatolicum* actin gene was taken as an endogenous control. The sequence of primers is provided in Table S3.

### Pull-down and MS with recombinant protein

To perform pull-down using recombinant protein, 96-well plate was coated with 1 μg/well of recombinant protein pHAMpin-1 and 1% BSA (used as a negative control) in bicarbonate/carbonate buffer incubated overnight at 4 °C. After incubation, wells were washed, blocked with 1% BSA, and incubated with 10% bovine serum in PBS for 30 min at 37 °C under agitation. Further, wells were washed, eluted with 0.2 M glycine, pH 2.3, and neutralized with 1 M Tris–HCl, pH 9. The samples were subjected to MS analysis as described above.

For the ELISA, complement proteins (C2, C4, C3, FH, and C5) were coated at 0.2 μg/well using the bicarbonate/carbonate buffer, followed by incubation with the eHAMpin-1 (1.5 μM). Detection was performed using HRP-conjugated anti-His antibody. For the Western blot analysis, eHAMpin-1 (5 μM) was incubated with 50 μl Ni-NTA beads (empty beads were used as a control), followed by overnight incubation with 10% NHS at 4 °C in binding buffer (20 mM Tris–HCl pH 8.0, 300 mM NaCl). Between each step, washing was performed using binding buffer. The samples were then eluted using elution buffer (100 mM imidazole, 20 mM Tris–HCl pH 8.0, 300 mM NaCl) and was analyzed in Western blot.

### Statistical analysis

Data are presented in all graphs as mean ± the standard deviation of the mean (SEM). Differences between the mean values of three or more groups were analyzed by one-way ANOVA. All statistical tests were conducted using GraphPad Prism 8.0.2 software (https://www.graphpad.com).

## Data availability

All data are contained within the manuscript. Datasets for *H. anatolicum* salivary gland long-read transcriptome data are submitted to BioProject; accession number: PRJNA1101950.

## supporting information

This article contains supporting information (11, 40, 43, 44, 45, 46, 64, 66, 67, 68, 69, 70, 71).

## Conflict of interest

The authors declare that they have no conflicts of interest with the contents of this article.

## Supporting information

Sheet S1

Sheet S2
