# Supplementary material for: The serine protease inhibitor HAMpin-1 produced by the ectoparasite Hyalomma anatolicum salivary gland modulates the host complement system
Source: J Biol Chem. 2024 Aug 17;300(9):107684. doi: 10.1016/j.jbc.2024.107684 (PMC11417211; doi:10.1016/j.jbc.2024.107684)
Supplement: Table S1 [file mmc3.docx]

**Table S1.** Superimposed structure of HAMpin-1 (Cyan) with resolved structure of *Ixodes ricinus* serpins (Tints wheat).

|  | **Resolved Serpin Structures** | **PDB ID** | **RMSD (Å)** | **Superimposed Structure** |
| --- | --- | --- | --- | --- |
| HAMpin-1  (*Hyalomma anatolicum*) | Iripin-1  (*Ixodes ricinus*) | 7QTZ | 1.281 | 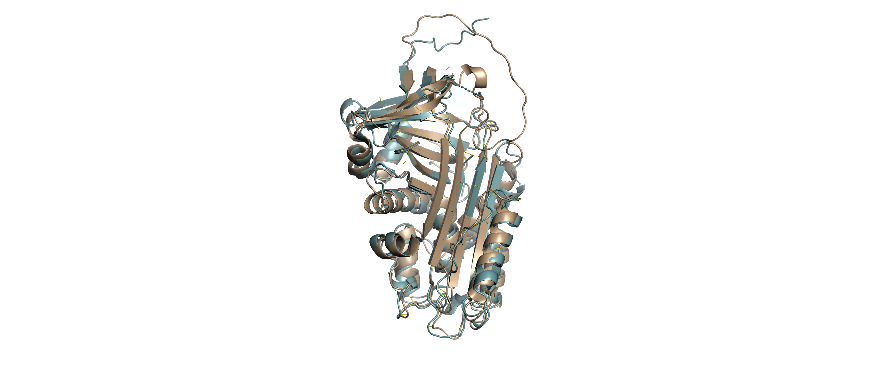 |
|  | Iripin-3  (*Ixodes ricinus*) | 7AHP | 1.520 | 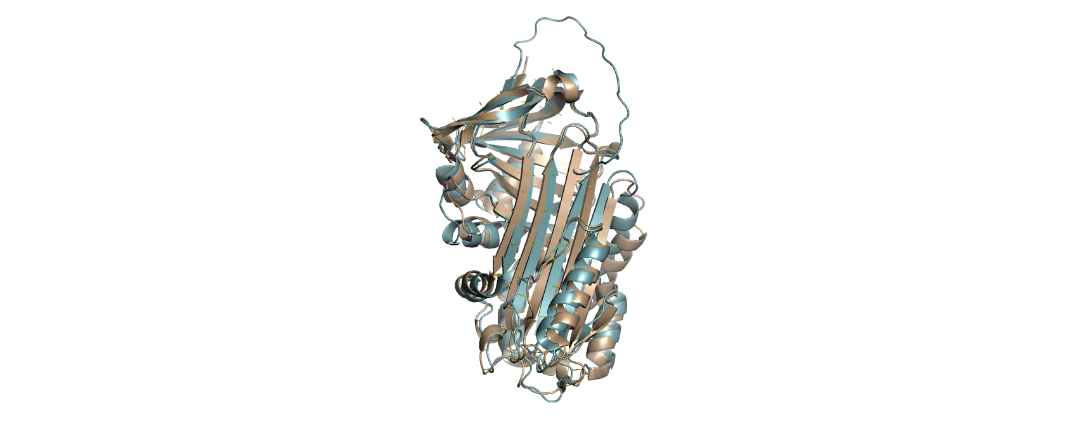 |
|  | Iripin-4  (*Ixodes ricinus*) | 7ZBF | 1.136 | 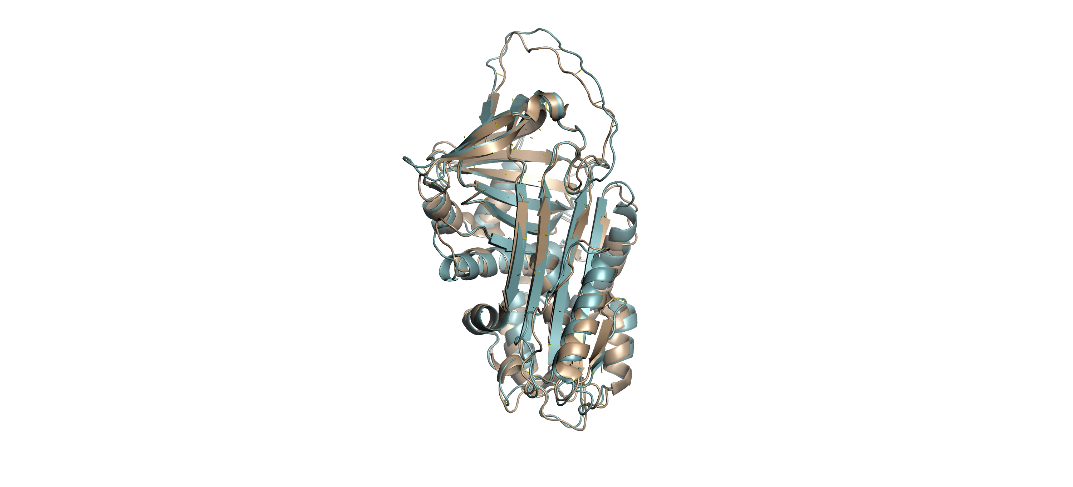 |
|  | IRS-2  (*Ixodes ricinus*) | 3NDA | 1.400 | 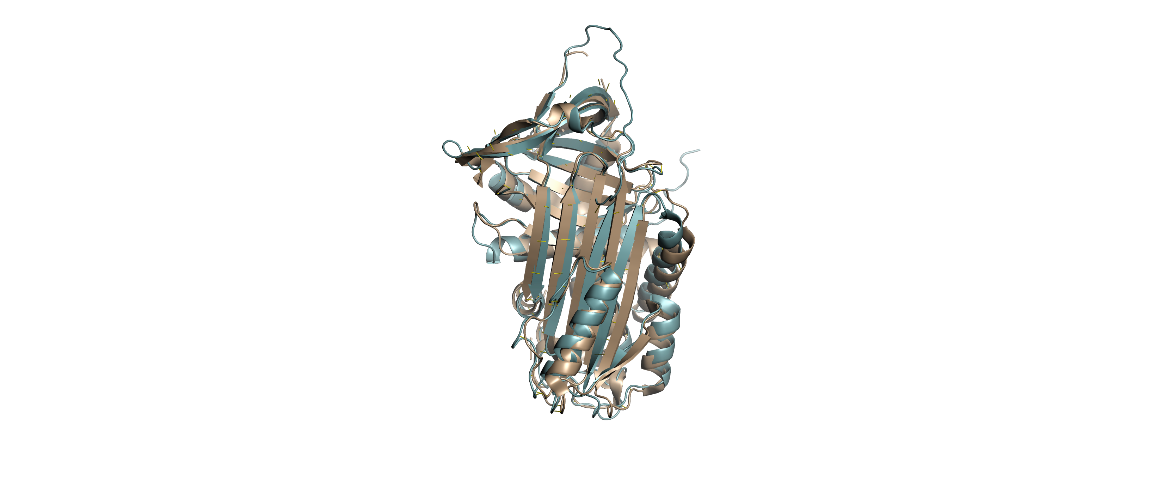 |
