# Supplementary material for: The serine protease inhibitor HAMpin-1 produced by the ectoparasite Hyalomma anatolicum salivary gland modulates the host complement system
Source: J Biol Chem. 2024 Aug 17;300(9):107684. doi: 10.1016/j.jbc.2024.107684 (PMC11417211; doi:10.1016/j.jbc.2024.107684)
Supplement: Table S2 [file mmc4.docx]

**Table S2.** Accession numbers of serpins used in the phylogenetic and RCL region comparison analysis.

| **Serpin** | **Accession number** | **Species** | **Reference** |
| --- | --- | --- | --- |
| AamS6 | ABS87358.1 | *Amblyomma americanum* | (1, 2) |
| AAS19 | JAI08902.1 |  |  |
| AAS27 | JAI08961.1 |  |  |
| AAS41 | JAI08957.1 |  |  |
| Ipis-1 | BAP59746.1 | *Ixodes persulcatus* | (3) |
| HLS2 | BAD11156.1 | *Haemaphysalis longicornis* | (4, 5) |
| HlSerpin-a | QFQ50847.1 |  |  |
| HlSerpin-b | QFQ50848.1 |  |  |
| Iripin-8 | 7PMU_A | *Ixodes ricinus* | (6-9) |
| Iripin-3 | JAA69032.1 |  |  |
| Iris | CAB55818.2 |  |  |
| IRS-2 | ABI94056.2 |  |  |
| IxscS-1E1 | AID54718.1 | *Ixodes scapularis* | (10) |
| RAS-1 | AAK61375.1 | *Rhipicephalus appendiculatus* | (11) |
| RAS-2 | AAK61376.1 |  |  |
| RAS-3 | AAK61377.1 |  |  |
| RAS-4 | AAK61378.1 |  |  |
| RHS-1 | AFX65224.1 | *Rhipicephalus haemaphysaloides* | (12, 13) |
| RHS-2 | AFX65225.1 |  |  |
| RHS8 | QHU78941.1 |  |  |
| RmS-1 | AHC98652.1 | *Rhipicephalus microplus* | (14) |
| RmS-3 | AHC98654.1 |  |  |
| RmS-6 | AHC98657.1 |  |  |
| RmS-15 | AHC98666.1 |  |  |
| RmS-17 | AHC98668.1 |  |  |
| A1AT | AAB59495.1 | *Homo sapiens* | (15) |

References:

1. Porter, L., Radulovic, Z., Kim, T., Braz, G. R., Da Silva Vaz, I., Jr., andMulenga, A. (2015) Bioinformatic analyses of male and female Amblyomma americanum tick expressed serine protease inhibitors (serpins) Ticks Tick Borne Dis **6**, 16-30 10.1016/j.ttbdis.2014.08.002

2. Mulenga, A., Khumthong, R., andBlandon, M. A. (2007) Molecular and expression analysis of a family of the Amblyomma americanum tick Lospins J Exp Biol **210**, 3188-3198 10.1242/jeb.006494

3. Toyomane, K., Konnai, S., Niwa, A., Githaka, N., Isezaki, M., Yamada, S. *et al.* (2016) Identification and the preliminary in vitro characterization of IRIS homologue from salivary glands of Ixodes persulcatus Schulze Ticks Tick Borne Dis **7**, 119-125 10.1016/j.ttbdis.2015.09.006

4. Imamura, S., da Silva Vaz Junior, I., Sugino, M., Ohashi, K., andOnuma, M. (2005) A serine protease inhibitor (serpin) from Haemaphysalis longicornis as an anti-tick vaccine Vaccine **23**, 1301-1311 10.1016/j.vaccine.2004.08.041

5. Wang, F., Song, Z., Chen, J., Wu, Q., Zhou, X., Ni, X. *et al.* (2020) The immunosuppressive functions of two novel tick serpins, HlSerpin-a and HlSerpin-b, from Haemaphysalis longicornis Immunology **159**, 109-120 10.1111/imm.13130

6. Kotal, J., Polderdijk, S. G. I., Langhansova, H., Ederova, M., Martins, L. A., Berankova, Z. *et al.* (2021) Ixodes ricinus Salivary Serpin Iripin-8 Inhibits the Intrinsic Pathway of Coagulation and Complement Int J Mol Sci **22**, 10.3390/ijms22179480

7. Chlastakova, A., Kotal, J., Berankova, Z., Kascakova, B., Martins, L. A., Langhansova, H. *et al.* (2021) Iripin-3, a New Salivary Protein Isolated From Ixodes ricinus Ticks, Displays Immunomodulatory and Anti-Hemostatic Properties In Vitro Front Immunol **12**, 626200 10.3389/fimmu.2021.626200

8. Leboulle, G., Crippa, M., Decrem, Y., Mejri, N., Brossard, M., Bollen, A. *et al.* (2002) Characterization of a novel salivary immunosuppressive protein from Ixodes ricinus ticks The Journal of biological chemistry **277**, 10083-10089 10.1074/jbc.M111391200

9. Chmelar, J., Oliveira, C. J., Rezacova, P., Francischetti, I. M., Kovarova, Z., Pejler, G. *et al.* (2011) A tick salivary protein targets cathepsin G and chymase and inhibits host inflammation and platelet aggregation Blood **117**, 736-744 10.1182/blood-2010-06-293241

10. Ibelli, A. M., Kim, T. K., Hill, C. C., Lewis, L. A., Bakshi, M., Miller, S. *et al.* (2014) A blood meal-induced Ixodes scapularis tick saliva serpin inhibits trypsin and thrombin, and interferes with platelet aggregation and blood clotting International journal for parasitology **44**, 369-379 10.1016/j.ijpara.2014.01.010

11. Mulenga, A., Tsuda, A., Onuma, M., andSugimoto, C. (2003) Four serine proteinase inhibitors (serpin) from the brown ear tick, Rhiphicephalus appendiculatus; cDNA cloning and preliminary characterization Insect biochemistry and molecular biology **33**, 267-276 10.1016/s0965-1748(02)00240-0

12. Yu, Y., Cao, J., Zhou, Y., Zhang, H., andZhou, J. (2013) Isolation and characterization of two novel serpins from the tick Rhipicephalus haemaphysaloides Ticks Tick Borne Dis **4**, 297-303 10.1016/j.ttbdis.2013.02.001

13. Xu, Z., Yan, Y., Zhang, H., Cao, J., Zhou, Y., Xu, Q. *et al.* (2020) A serpin from the tick Rhipicephalus haemaphysaloides: Involvement in vitellogenesis Veterinary parasitology **279**, 109064 10.1016/j.vetpar.2020.109064

14. Tirloni, L., Seixas, A., Mulenga, A., Vaz Ida, S., Jr., andTermignoni, C. (2014) A family of serine protease inhibitors (serpins) in the cattle tick Rhipicephalus (Boophilus) microplus Exp Parasitol **137**, 25-34 10.1016/j.exppara.2013.12.001

15. Long, G. L., Chandra, T., Woo, S. L., Davie, E. W., andKurachi, K. (1984) Complete sequence of the cDNA for human alpha 1-antitrypsin and the gene for the S variant Biochemistry **23**, 4828-4837 10.1021/bi00316a003
