# Supplementary material for: The serine protease inhibitor HAMpin-1 produced by the ectoparasite Hyalomma anatolicum salivary gland modulates the host complement system
Source: J Biol Chem. 2024 Aug 17;300(9):107684. doi: 10.1016/j.jbc.2024.107684 (PMC11417211; doi:10.1016/j.jbc.2024.107684)
Supplement: Table S3 [file mmc5.docx]

| **Primer name** | **Primer sequence (5’ to 3’)** | **Vector** |
| --- | --- | --- |
| **Cloning primers** | | |
| HAMpin-1_FP | GGAATTCCATATGTCAGAGACGATGGCTTCCAACC | pET21a |
| HAMpin-1_RP | ATAAGAATGCGGCCGCAAGTTGGCGAACAGACCCCATG |  |
| HAMpin-1_pPICZαA_FP | CGGGGTACCATGTCAGAGACGATGGCTTCCAACC | pPICZα-A |
| HAMpin-1_ pPICZαA_RP | GCTCTAGAGCAAGTTGGCGAACAGACCCCATG |  |
| **RT-PCR primers** | | |
| HAMpin-1_FP | ATGTCAGAGACGATGGCTTCC | - |
| HAMpin-1_RP | CGAGGTAGCTGTCCTGGA | - |
| Tick_Actin_FP | ATTGAGCACGGTATCGTCACC | - |
| Tick_Actin_RP | CGAGCACGATACCGGTGG | - |
| Sequencing Primer | | |
| 5’ AOX1 | GACTGGTTCCAATTGACAAGC |  |
| 3’ AOX1 | GCAAATGGCATTCTGACATCC |  |

**Table S3.** List of primers used in this study. Restriction sites underlined for cloning primers.

Supplementary tables Table S1. List of primers used in this study.
